# Dual-targeted magnetic mesoporous silica nanoparticles reduce brain amyloid-β burden via depolymerization and intestinal metabolism

**DOI:** 10.7150/thno.76574

**Published:** 2022-09-11

**Authors:** Ni Liu, Xiaohan Liang, Changwen Yang, Shun Hu, Qingming Luo, Haiming Luo

**Affiliations:** 1 Britton Chance Center for Biomedical Photonics, Wuhan National Laboratory for Optoelectronics, Huazhong University of Science and Technology, Wuhan, China.; 2 MoE Key Laboratory for Biomedical Photonics, School of Engineering Sciences, Huazhong University of Science and Technology, Wuhan, China.; 3 School of Biomedical Engineering, Hainan University, Haikou, Hainan 570228, China.

**Keywords:** Alzheimer's disease, amyloid-β (Aβ), magnetic mesoporous silica nanoparticles, Aβ clearance, Aβ_42_-targeting antibody.

## Abstract

**Rationale:** Active removal of excess peripheral amyloid-β (Aβ) can potentially treat Alzheimer's disease (AD). However, the peripheral clearance of Aβ using an anti-Aβ monoclonal antibody (mAb) cannot remove PET-detectable Aβ within the brain. This may be due to the inability of mAb to cross the blood-brain barrier (BBB) to degrade insoluble brain Aβ plaques and block liver dysfunction.

**Methods:** We developed a dual-targeted magnetic mesoporous silica nanoparticle (HA-MMSN-1F12) through surface-coupled Aβ_42_-targeting antibody 1F12 and CD44-targeting ligand hyaluronic acid (HA).

**Results:** HA-MMSN-1F12 had a high binding affinity toward Aβ_42_ oligomers (Kd = 1.27 ± 0.34 nM) and revealed robust degradation of Aβ_42_ aggregates. After intravenous administration of HA-MMSN-1F12 into ten-month-old APP/PS1 mice for three weeks (4 mg/kg/week), HA-MMSN-1F12 could cross the BBB and depolymerize brain Aβ plaques into soluble Aβ species. In addition, it also avoided hepatic uptake and excreted captured Aβ species through intestinal metabolism, thereby reducing brain Aβ load and neuroinflammation and improving memory deficits of APP/PS1 mice. Furthermore, the biochemical analysis showed that HA-MMSN-1F12 did not detect any toxic side effects on the liver and kidney. Thus, the efficacy of HA-MMSN-1F12 is associated with the targeted degradation of insoluble brain Aβ plaques, avoidance of non-specific hepatic uptake, and excretion of peripheral Aβ through intestinal metabolism.

**Conclusions:** The study provides a new avenue for treating brain diseases by excreting disease-causing biohazards using intestinal metabolism.

## Introduction

Alzheimer's disease (AD), a progressive and destructive disorder, is characterized by abnormal amyloid-β (Aβ) protein deposits in the brain 1. These neurotoxic Aβ peptides form insoluble filaments that accumulate into amyloid plaques in the brain and blood vessels. Abnormal Aβ accumulation precedes neurodegeneration and cognitive decline in familial and sporadic AD, indicating that abnormal Aβ brain metabolism has a central role in AD pathogenesis. Pathological Aβ deposition could be due to an altered balance between overproduction and elimination. Evidence indicates that active removal of excess peripheral Aβ could be a potential treatment strategy for AD 2. Immunotherapy with anti-Aβ antibodies is the most likely treatment strategy for disease management. Various peripheral interventions have been attempted, including Aβ vaccination, intravenous anti-Aβ antibody administration, and intravenous immunoglobulin administration, to slow down Aβ deposition and reduce AD. However, peripheral clearance of Aβ with the anti-Aβ monoclonal antibody (mAb) solanezumab in clinical studies cannot appear to remove PET-detectable Aβ from the brain, so alterations in peripheral Aβ metabolism may not be associated with AD 3. Many factors may cause the failure of solanezumab to remove Aβ deposits from the brain. However, the most likely reason is that once Aβ is sequestered in insoluble plaques, a simple concentration gradient might not dissolve the deposits. This could be due to immune complex formation with soluble Aβ in the blood after intravenous infusion of anti-Aβ monoclonal antibodies, with a half-life of days to weeks. In contrast, the half-life of free Aβ peptides is several hours. Therefore, effective strategies to remove insoluble Aβ aggregates from the brain will be the key to reducing the brain Aβ burden.

Aβ clearance may also depend on the entry of anti-Aβ antibodies into the brain, but only about 0.1-0.2% of peripherally injected antibodies can enter 4. Various strategies, including focused ultrasound 5, intranasal administration 6, or multiple formulations 7, can increase the uptake of antibodies and biomacromolecules in the brain. Receptor-mediated transcytosis is currently the most commonly used strategy for delivering antibodies to the brain. In this approach, antibodies are fused to antibodies targeting transferrin 8 or insulin receptors 9 located on the surface of BBB-forming endothelial cells. Transferrin receptor (TfR) and low-density lipoprotein receptor-related protein-1 (LRP1) enhance brain uptake of antibodies through antibody fusions 10-14 or bispecific constructs 15, 16. However, TfR and LRP1 are widely expressed in cells of various tissue, including brain endothelial cells, hepatocytes, and tumor cells, which would increase the non-specific accumulation of TfR-modifying drugs in these tissues, especially in the liver. Furthermore, strategies for drug delivery to the brain using anti-TfR antibodies suffer from low specificity to brain tissue and immunogenicity 15, 17, 18.

Hyaluronic acid (HA) is a biodegradable, biocompatible, and non-immunogenic glycosaminoglycan that is recognized by the CD44 receptor on the surface of endothelial cells 19. Although HA targets CD44-overexpressing tumor cells 20, it can mediate its conjugates across the BBB via endocytosis. Nanomaterials retain the properties of antibodies and offer better design flexibility and functionality to combine therapeutic agents in one structure 21. However, anti-Aβ mAb-modified liposomes 22, polymeric nanoparticles 23, inorganic nanoparticles 24, 25, and biomimetic nanozymes 26 can clear peripheral Aβ in AD transgenic mice. The administered nanomaterials accumulate in the liver, resulting in Aβ accumulation being captured by the nanomaterials. In contrast, rodent studies have shown impaired hepatic Aβ degradation in AD subjects 27. The abundance of these toxic and non-degradable Aβ may be deposited in the liver, accelerating the pathological process of AD 28. Therefore, overcoming liver dysfunction ensures the targeted clearance of insoluble Aβ in the brain and reduces its Aβ burden.

The accumulation of nanodrugs in the liver is regulated by their size, charge, surface modification, and their choice 29. Mesoporous silica nanoparticles (MSNs) are relatively safe and porous, with good biocompatibility and easy surface modification. MSN particles larger than 100 nm depicted low cytotoxicity, while particles smaller than 50 nm significantly induce cell necrosis 30, 31. Based on recent findings and our previously developed anti-Aβ_42_ monoclonal antibodies (mAbs) 32, we synthesized 413 nm functionalized magnetic mesoporous silica nanospheres with surface-bound anti-Aβ mAb 1F12 and CD44-targeted HA ligands (HA-MMSN-1F12) to evaluate its ability to depolymerize Aβ aggregates into monomers *in vitro*. In addition, APP/PS1 transgenic mice were used to evaluate the ability of HA-MMSN-1F12 to cross the BBB and its biodistribution and therapeutic effects. This innovative concept of using nanotechnology to solve the problem of AD liver dysfunction and the inability of mAbs to enter the brain to degrade insoluble brain Aβ plaques opens up a new avenue for AD treatment.

## Results

### Characterization of HA-MMSN-1F12 with dual targeting of Aβ_42_ and CD44

**Figure 1:**
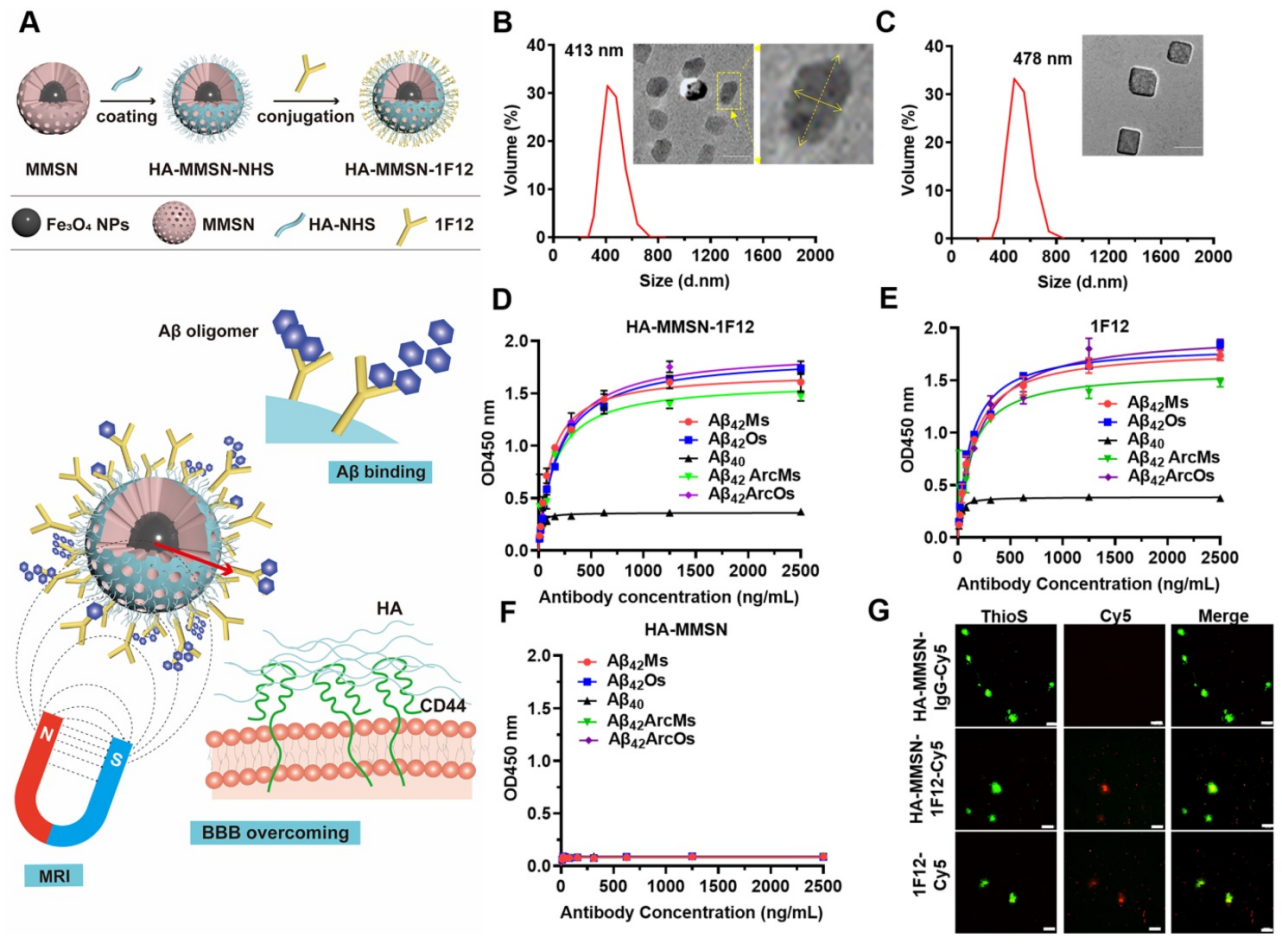
** Synthesis and characterization of HA-MMSN-1F12. (A)** Schematic diagram of HA-MMSN-1F12 synthesis. Particle size and TEM images of **(B)** HA-MMSN and **(C)** HA-MMSN-1F12, aspect ratio 2. The binding affinity of **(D)** HA-MMSN-1F12, **(E)** 1F12, and **(F)** HA-MMSN is based on different Aβ conformations. **(G)** Confocal imaging of brain sections from eight-month-old APP/PS1 mice incubated with Cy5-labeled 1F12, HA-MMSN-1F12, and HA-MMSN-IgG and counterstained using ThioS. Cy5 (red), ThioS (green), and scale bar, 50 μm.

A dual-targeted Aβ clearance system based on MMSN was constructed to achieve efficient clearance of Aβ in the brain. The anti-Aβ_42_-targeting antibody 1F12 was introduced to capture Aβ_42_ peptides, and the surface-coupled CD44-targeting ligand HA efficiently enhanced the brain entry of HA-MMSN-1F12 (Figure 1A). HA-MMSN-1F12 was characterized for size, shape, hydrodynamic diameter, zeta potential, and binding affinity for Aβ species. After surface modification, the hydrodynamic diameter of HA-MMSN-1F12 ranged from 413 to 478 nm. Moreover, the PDI value decreased from 0.049 to 0.045, supporting the improved stability of HA-MMSN-1F12 (Figure 1B-C). HA-MMSN-1F12 depicted a negative zeta potential of -13.03 ± 0.12 mV (Figure S1A). Fourier transform infrared (FT-IR) spectroscopy, SDS-PAGE, and Western blotting confirmed that 1F12 was successfully immobilized on the MMSN surface (Figure S1B-C). Furthermore, HA-MMSN-1F12 had good stability of HA-MMSN-1F12 in plasma with less adsorption to serum albumin and no specificity (Figure S3). ELISA and IP-Western blotting data revealed that HA-MMSN-1F12 retained the characteristics of 1F12 and could specifically recognize soluble Aβ_42_ and Aβ_42_Arc, monomers, and oligomers, but not Aβ_40_ (Figure S4A-B). The core of the MMSN consists of numerous magnetic nanoparticles generating nuclear magnetic signals via magnetic resonance imaging (MRI). As shown in Figure S2, the T2 relaxation time of the MMSN is 94.89 ms, and the saturation recovery (SR) pulse showed that the MMSN had a good T1 mapping. These results indicated that the MMSN had a good MRI signal.

In addition, the minimum detection limit of Aβ_42_Os was 0.5 pM, providing excellent sensitivity for the detection of peripheral and central Aβ_42_ (Figure S5). Indirect ELISA depicted that the K_d_ values of HA-MMSN-1F12 for binding to Aβ_42_ were 0.77 ± 0.09 nM for Aβ_42_Ms, 1.27 ± 0.34 nM for Aβ_42_Os, 0.98 ± 0.17 nM for Aβ_42_ArcMs, and 1.27 ± 0.26 nM for Aβ_42_ArcOs, respectively, comparable to the observed 1F12 values (0.95 ± 0.01 nM, 0.82 ± 0.01 nM, 0.85 ± 0.18 nM, and 1.31 ± 0.17 nM, respectively) (Figure 1D-E). In contrast, HA-MMSN showed no affinity toward different Aβ_42_ conformations (Figure 1F). Immunofluorescence imaging of brain sections from eight-month-old APP/PS1 mice revealed that ThioS-positive Aβ plaques colocalized with Cy5-labeled 1F12 and HA-MMSN-1F12, but not with HA-MMSN-IgG (Figure 1G and Figure S4C), depicting the binding specificity of HA-MMSN-1F12 toward Aβ plaques. Therefore, HA-MMSN-1F12 showed a preference for capturing Aβ_42_ species rather than Aβ_40_.

### Assessing the ability of HA-MMSN-1F12 to depolymerize insoluble Aβ_42_ aggregates

**Figure 2:**
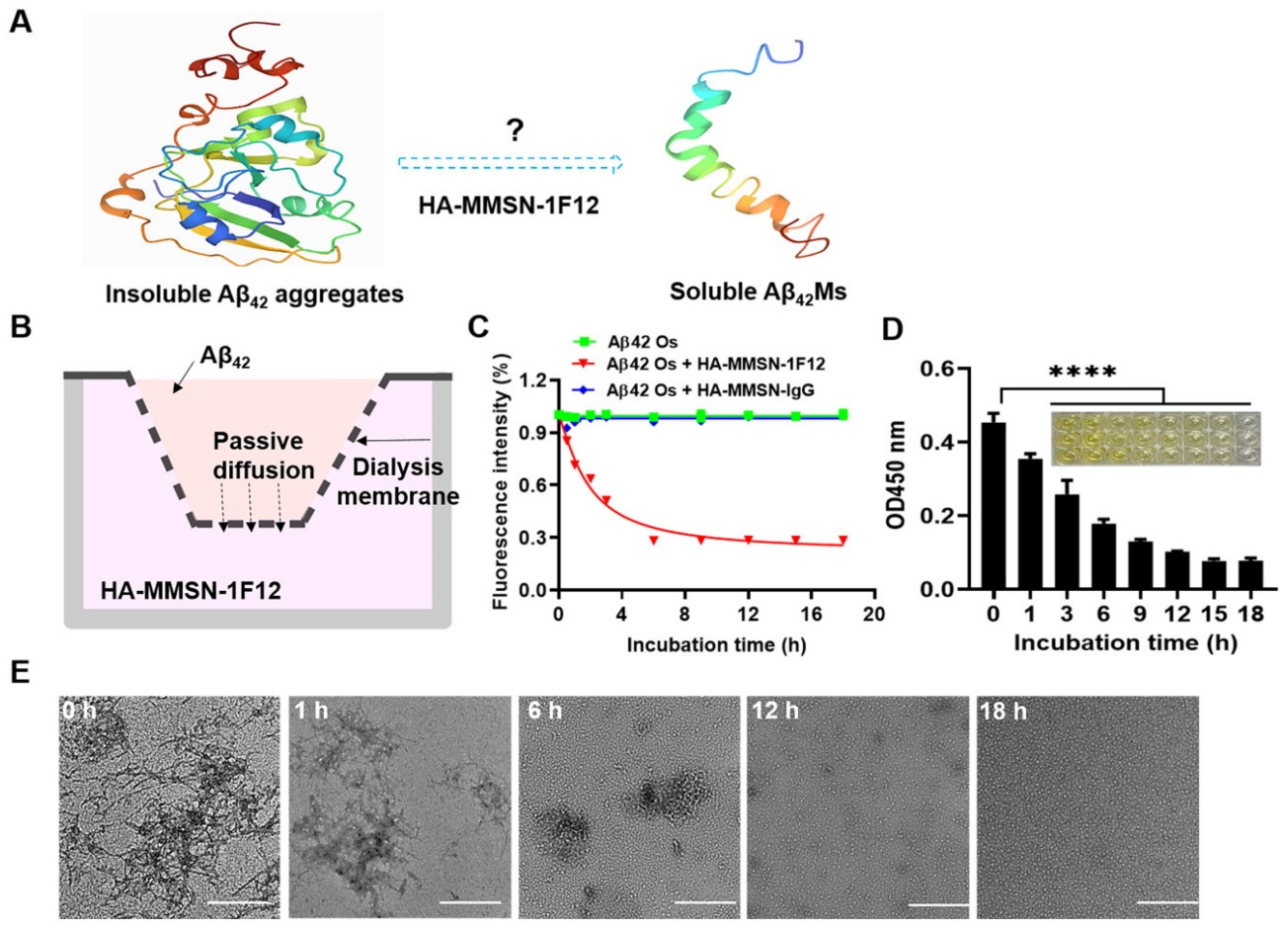
** Evaluating the effect of HA-MMSN-1F12 on Aβ_42_ aggregate degradation. (A)** Schematic diagrams of degrading Aβ_42_ aggregates to monomers. **(B)** Construction of an *in vitro* Aβ_42_ aggregate degradation system using the transwell system. **(C)** The degradation kinetics of Aβ_42_Os in the upper chamber under the HA-MMSN-1F12 or HA-MMSN-IgG action in the lower chamber was monitored through ThT fluorescence.** (D)** ELISA was used to monitor changes in the level of Aβ_42_Os in the upper chamber under the action of HA-MMSN-1F12. **(E)** TEM images of Aβ_42_ in the upper chamber when Aβ_42_Os are in the upper chamber were incubated using HA-MMSN-1F12 in the lower chamber at 37 °C for 0, 1, 6, 12, and 18 h. Scale bar, 500 nm.

A system for degrading Aβ_42_ aggregates *in vitro* was constructed to investigate whether HA-MMSN-1F12 could degrade Aβ_42_ aggregates into monomers. If Aβ_42_ aggregates in the upper chamber can be depolymerized into monomers, due to the difference in Aβ_42_ concentration, Aβ_42_ monomers (Aβ_42_Ms) will be transferred to the lower chamber and captured by HA-MMSN-1F12 (Figure 2A-B). In addition, ThT fluorescence and ELISA assays were performed to quantify changes in Aβ_42_ oligomers (Aβ_42_Os) levels in the upper chamber after different treatments. The results showed that the ThT fluorescence intensity in the upper chamber of the HA-MMSN-1F12-treated group was significantly lower than that of the HA-MMSN-IgG-treated group (Figure 2C), which was consistent with the ELISA results (Figure 2D). This indicated that Aβ_42_Os in the upper chamber were continuously dissociated into Aβ_42_Ms under the action of HA-MMSN-1F12 in the lower chamber, resulting in a gradual decrease in the level of Aβ_42_Os in the upper chamber.

Next, the morphological changes of Aβ_42_ aggregates disaggregated in the upper chamber at different times under the action of HA-MMSN-1F12 in the lower chamber were confirmed by TEM. Figure 2E shows that under the action of HA-MMSN-1F12, the number of Aβ_42_ aggregates in the upper chamber continued to decrease and the number of Aβ_42_Ms gradually increased. After more than 18 h of incubation with HA-MMSN-1F12 in the lower chamber, only Aβ_42_Ms were present in the upper chamber. In addition, HA-MMSN-1F12 also inhibited the self-assembly of Aβ_42_ aggregates (Figure S6). Therefore, HA-MMSN-1F12 could promote the disaggregation of Aβ_42_ aggregates and inhibit their self-assembly.

### Evaluation of brain delivery efficiency and intestinal metabolism of HA-MMSN-1F12

**Figure 3:**
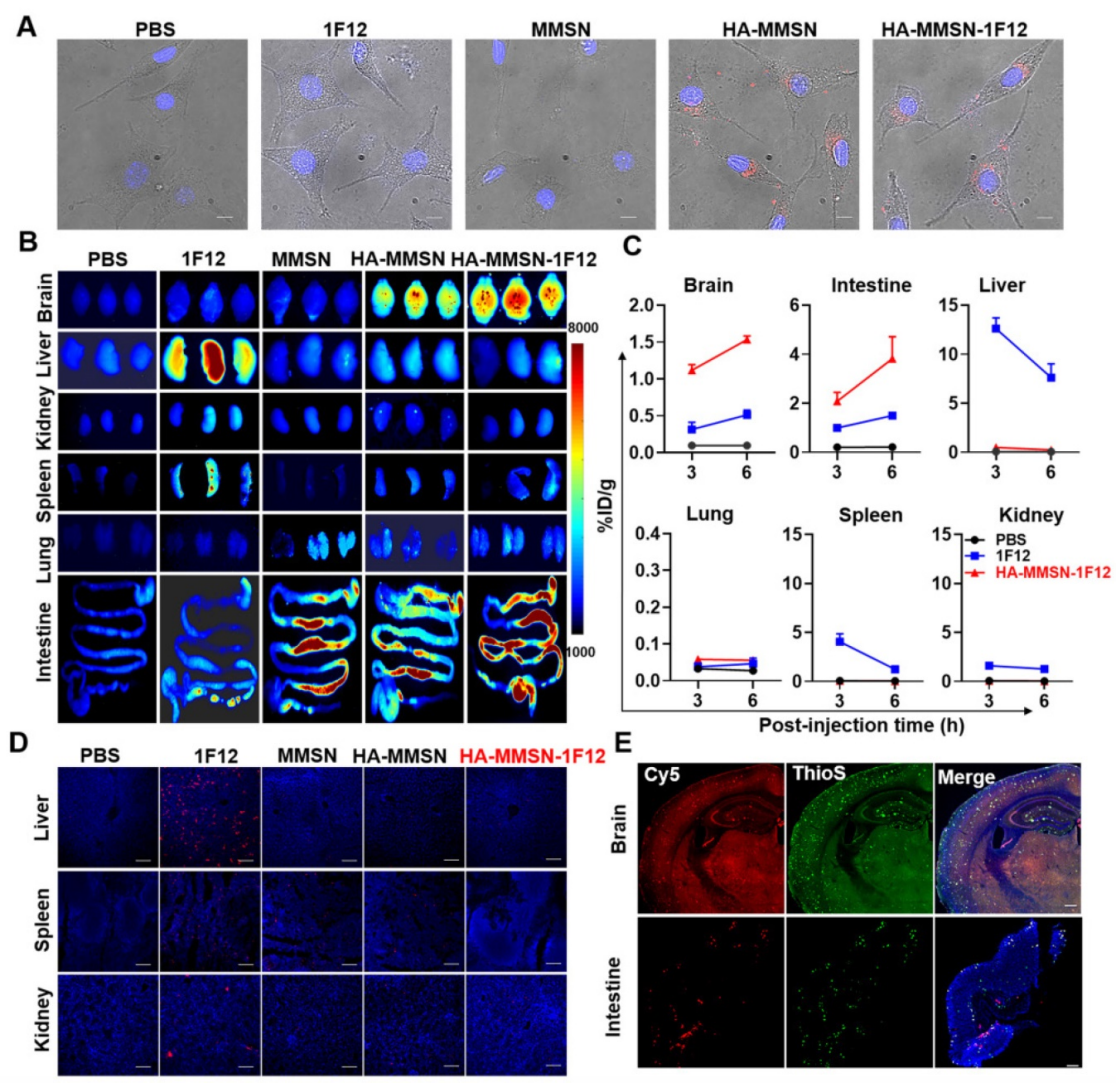
**Evaluation of brain delivery efficiency and intestinal metabolism of HA-MMSN-1F12. (A)** Confocal imaging of the SH-SY5Y cells in the transwell system and pretreated using Cy3-labeled 1F12, MMSN, HA-MMSN-1F12, or HA-MMSN in the upper chamber for evaluating the ability of these fluorescent probes to enter the brain*.* DAPI (blue), Cy3 (red), and scale bar, 200 µm. **(B)** Fluorescence imaging of the organs from ten-month-old APP/PS1 mice at 3 h post-injection of the Cy5-labeled 1F12, MMSN, HA-MMSN-1F12, and HA-MMSN. n = 3 per group. **(C)** Quantitative analysis of the organs homogenates from ten-month-old APP/PS1 mice at 3 and 6 h post-injection of Cy5-labeled 1F12, MMSN, HA-MMSN-1F12, and HA-MMSN. n = 3 per group. **(D)** Confocal imaging of liver, spleen, and kidney tissue sections from eight-month-old APP/PS1 mice at 3 h p.i. of Cy5-labeled 1F12, MMSN, HA-MMSN-1F12, and HA-MMSN; DAPI (blue), Cy5 (red), and the scale bar, 100 µm; n = 3 per group.** (E)** Confocal imaging of the brain and intestinal tissue sections from the ten-month-old APP/PS1 mice at 6 h post-injection of Cy5-labeled HA-MMSN-1F12. DAPI (blue), Cy5 (red), ThioS (green), and scale bar, 200 µm. n = 3 per group.

HUVECs were incubated with Cy3-labeled 1F12, HA-MMSN-1F12, HA-MMSN, and MMSN in the upper chamber at 37 °C for 24 h to investigate their uptake by SY5Y cells in the lower chamber. Confocal imaging revealed a robust Cy3 signal detectable in SY5Y cells in the HA-MMSN and HA-MMSN-1F12-treated groups (Figure 3A). This indicated that HA could facilitate the passage of its conjugated nanoparticles through vascular endothelial cells. In addition, CD44 was highly expressed in ten-month-old APP/PS1 brains (Figure S8), which was more favorable for helping HA-MMSN-1F12 to cross the BBB.

Next, the efficiency of intravenous injection of HA-MMSN-1F12 across the BBB was investigated. APP/PS1 transgenic mice are an age-dependent developmental AD mouse model for brain Aβ deposition. After intravenous injection of Cy5-labeled 1F12, HA-MMSN-1F12, HA-MMSN, and MMSN in APP/PS1 mice, organ fluorescence imaging revealed strong fluorescence signals in the brains of mice treated with Cy5-labeled HA-MMSN and HA-MMSN-1F12 after 3 h of injection. In contrast, no signal was detected in the MMSN- and 1F12-treated groups (Figure 3B). Quantitative analysis of homogenized brain tissue revealed efficient accumulation of Cy5-labeled HA-MMSN-1F12 in the brain over time. Signals detected in the brain were 1.12 ± 0.08 and 1.57 ± 0.06 %ID/g at 3 and 6 h post-injection, respectively (Figure 3C). However, only 0.32 ± 0.10 and 0.51 ± 0.07 %ID/g signals were detected at 3 and 6 h post-injection for 1F12-treated mice, significantly lower (*p* < 0.0001) than those in HA-MMSN-1F12-treated mice, indicating that HA facilitates its conjugate across the BBB.

In addition, organ fluorescence imaging depicted a strong fluorescence signal in the liver of mice treated with Cy5-labeled 1F12 at 3 h post-injection. In contrast, no signal was detected in the MMSN- and PBS-treated groups (Figure 3B). According to a quantitative analysis of homogenized liver tissues, 12.60 ± 1.11 %ID/g and 7.62 ± 1.40 %ID/g signals were detected in mouse livers after 3 and 6 h post-injection of 1F12, respectively (Figure 3C). In contrast, only 0.07-0.50 %ID/g signal was detected in the liver of mice in the HA-MMSN-1F12-treated group, and the low liver uptake of HA-MMSN-1F12 was further confirmed by confocal imaging of tissue slices (Figure 3D). Furthermore, fluorescence imaging also revealed that Cy5-labeled MMSN, HA-MMSN, and HA-MMSN-1F12 were rapidly metabolized in the intestine after 3 h of injection, but not Cy5-1F12 (Figure 3B and Figure S9A). The uptake of Cy5-HA-MMSN-1F12 in the colon was 3.82 ± 0.89 %ID/g after 6 h of injection, which was significantly higher (*p* < 0.0001) than that of Cy5-1F12 (1.50 ± 0.11 %ID/g; Figure 3C and Figure S9A). This may be due to the shorter blood half-life of MMSN-based probes than Cy5-1F12 (Figure S9B). In addition, 3 h after intravenous administration of Cy5-HA-MMSN-1F12, weak fluorescence signals were also observed in the spleen, lung, and kidney of mice (Figure 3B-C). Immunofluorescence imaging of tissue sections revealed that the Cy5 fluorescence signal of HA-MMSN-1F12 but not HA-MMSN colocalized with the ThioS signal in the brain and small intestine (Figure 3D and Figure S10). The signals indicated that intravenously injected HA-MMSN-1F12 could specifically label Aβ in the brain via the BBB and that HA-MMSN-1F12 metabolized in the intestine could also target intestinal Aβ.

### Escape of HA-MMSN-1F12 uptake by Kupffer cells

**Figure 4:**
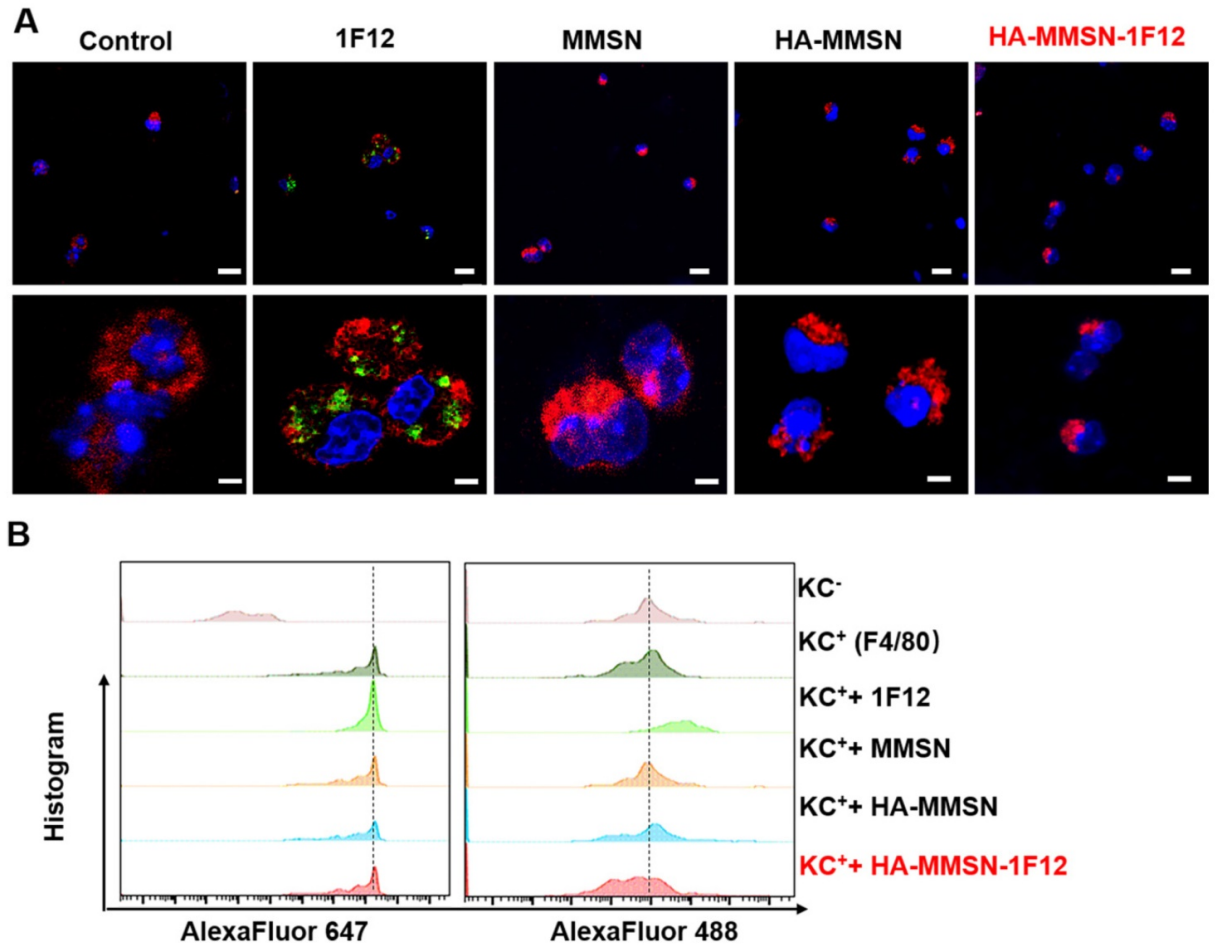
** Escape from the uptake of HA-MMSN-1F12 through KCs. (A)** Confocal imaging of KCs after 2 h incubation through the Alexa Fluor 488-labeled 1F12, MMSN, HA-MMSN-1F12, and HA-MMSN. **(B)** Representative flow cytometry histograms of KCs (F4/80-Alexa Fluor 647-positive) absorbed Alexa Fluor 488-labeled 1F12, MMSN, HA-MMSN-1F12, and HA-MMSN; DAPI (blue), F4/80-Alexa Fluor 647 (red), Alexa Fluor 488 (green). One-way analysis of variance (ANOVA) was utilized to undergo multigroup comparisons.

Kupffer cells (KCs) are resident hepatic macrophages responsible for the phagocytosis of circulating pathogens and exogenous nanomaterials. Next, the underlying mechanism leading to the good performance of HA-MMSN-1F12 was investigated. Alexa Fluor488-labeled 1F12, MMSN, HA-MMSN, and HA-MMSN-1F12 were incubated using AlexaFluor 647-F4/80 positive KCs (F4/80^+^-KCs) to understand the low liver absorption rate of HA-MMSN-1F12, marked as F4/80 to ensure primary KCs 33. Flow cytometry and confocal imaging showed that F4/80^+^-KC mainly occupied 1F12. In contrast, the uptake of MMSN, HA-MMSN, and HA-MMSN-1F12 by F4/80^+^-KC was negligible, suggesting that HA-MMSN-1F12 could avoid the uptake of KCs (Figure 4). This is extremely important in the clearance of peripheral Aβ_42_ by altering the metabolic pathway of circulating Aβ_42_ in the intestine.

### Evaluating the effect of HA-MMSN-1F12 on Aβ_42_ clearance in peripheral blood

**Figure 5:**
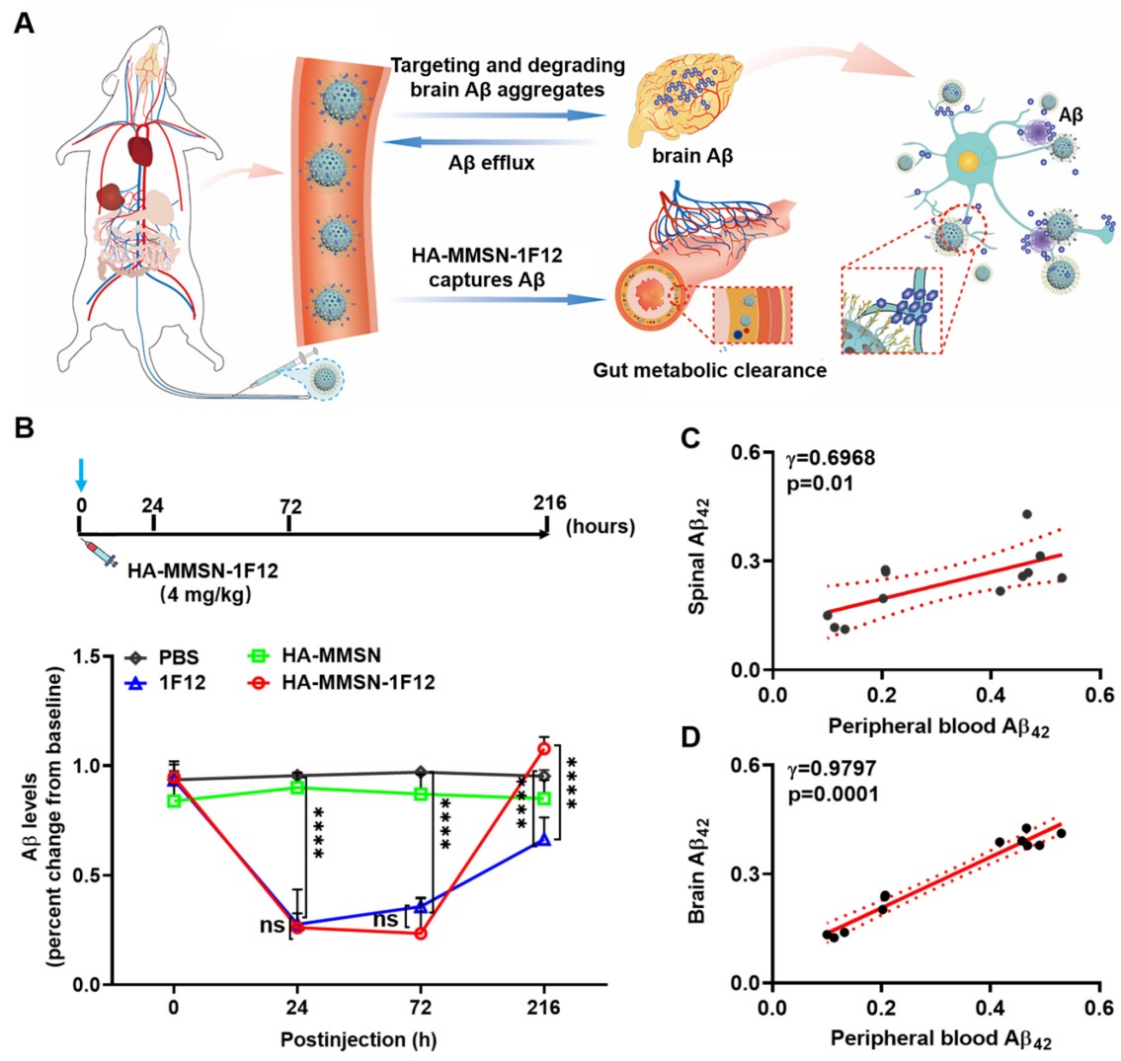
** Aβ clearance after the intravenous administration of HA-MMSN-1F12. (A)** Schematic illustrations of the Aβ burden reduction within the brain by intravenously injecting HA-MMSN-1F12. Intravenously injected HA-MMSN-1F12 crossed the blood-brain barrier into the brain for degrading insoluble Aβ aggregates into soluble monomers and flowing into the periphery. Similarly, peripheral soluble Aβ was captured using HA-MMSN-1F12 and excreted using intestinal metabolism, thereby reducing the brain Aβ burden. **(B)** Changes in the peripheral blood of Aβ levels in ten-month-old APP/PS1 mice after single-dose intravenous injection of HA-MMSN, 1F12, and HA-MMSN-1F12. The correlation between changes in Aβ levels in peripheral blood and spinal cord **(C)** or brain **(D)** (Pearson correlation). n = 3 per group. One-way analysis of variance (ANOVA) was performed to undergo multiple group comparisons. Statistical significance is considered with ^***^p < 0.001, and n.s (indicating no significance).

Intravenously injected HA-MMSN-1F12 could target and depolymerize Aβ brain plaques as monomers through the BBB and peripheral efflux. In contrast, all peripheral Aβ_42_ species would be captured by HA-MMSN-1F12 and excreted through intestinal metabolism, thereby reducing Aβ burden within the brain (Figure 5A). Mouse blood was incubated with protein A columns to enrich HA-MMSN-1F12 complexes and circulating Aβ_42_ 12 h after post-injection of HA-MMSN-1F12 to evaluate whether 4 mg/kg of HA-MMSN-1F12 could be an effective treatment dose. Western blotting results showed that distinct Aβ_42_Os bands were observed in protein A beads, but not in the protein A elution solution, indicating that HA-MMSN-1F12 captured all free Aβ_42_ in the blood (Figure S11A). Therefore, HA-MMSN-1F12 behaves as a sink for Aβ_42_ in the blood, and an intravenous dose of 4 mg/kg of HA-MMSN-1F12 can effectively bind all Aβ_42_ species.

Next, the effect of HA-MMSN-1F12 on Aβ_42_ clearance in peripheral blood and the dynamic interaction between peripheral and central Aβ_42_ levels was also investigated. Ten-month-old male APP/PS1 mice with the closest positive serum OD450 values were selected for the experiments to reduce errors due to differences between mice (Figure S11B). After a single intravenous dose of HA-MMSN-1F12, a decrease in peripheral Aβ_42_ levels was observed, reaching a minimum level 24 h after post-injection (*p* < 0.0001). Furthermore, it gradually increased and reached near baseline levels (Figure 5B). In contrast, Aβ_42_ levels in the blood of mice treated with HA-MMSN and PBS did not change significantly. Moreover, intravenous administration of IF12 significantly reduced Aβ_42_ levels in peripheral blood. Peripheral blood Aβ_42_ levels at 24 h (*p* = 0.9943) and 72 h (*p* > 0.1836) after post-injection were not significantly different from the HA-MMSN-1F12-treated group. However, peripheral blood Aβ_42_ levels in HA-MMSN-1F12-treated mice recovered significantly faster than in the 1F12-treated mice (*p* < 0.0001) and exceeded baseline levels (*p* = 0.2061) 216 h after injection (Figure 5B). In addition, regression analysis showed that soluble Aβ_42_ blood levels were positively correlated with those in the spinal cord (r = 0.6968; Figure 5C) and brain (r = 0.9797; Figure 5D), implying that more soluble Aβ_42_ was produced in the brain at 216 h post-injection. These results indicated that HA-MMSN-1F12 could enter the brain to depolymerize Aβ plaques and form more soluble Aβ to flow into the periphery, increasing peripheral blood Aβ levels.

### Intravenous administration of HA-MMSN-1F12 improves cognitive deficits

**Figure 6:**
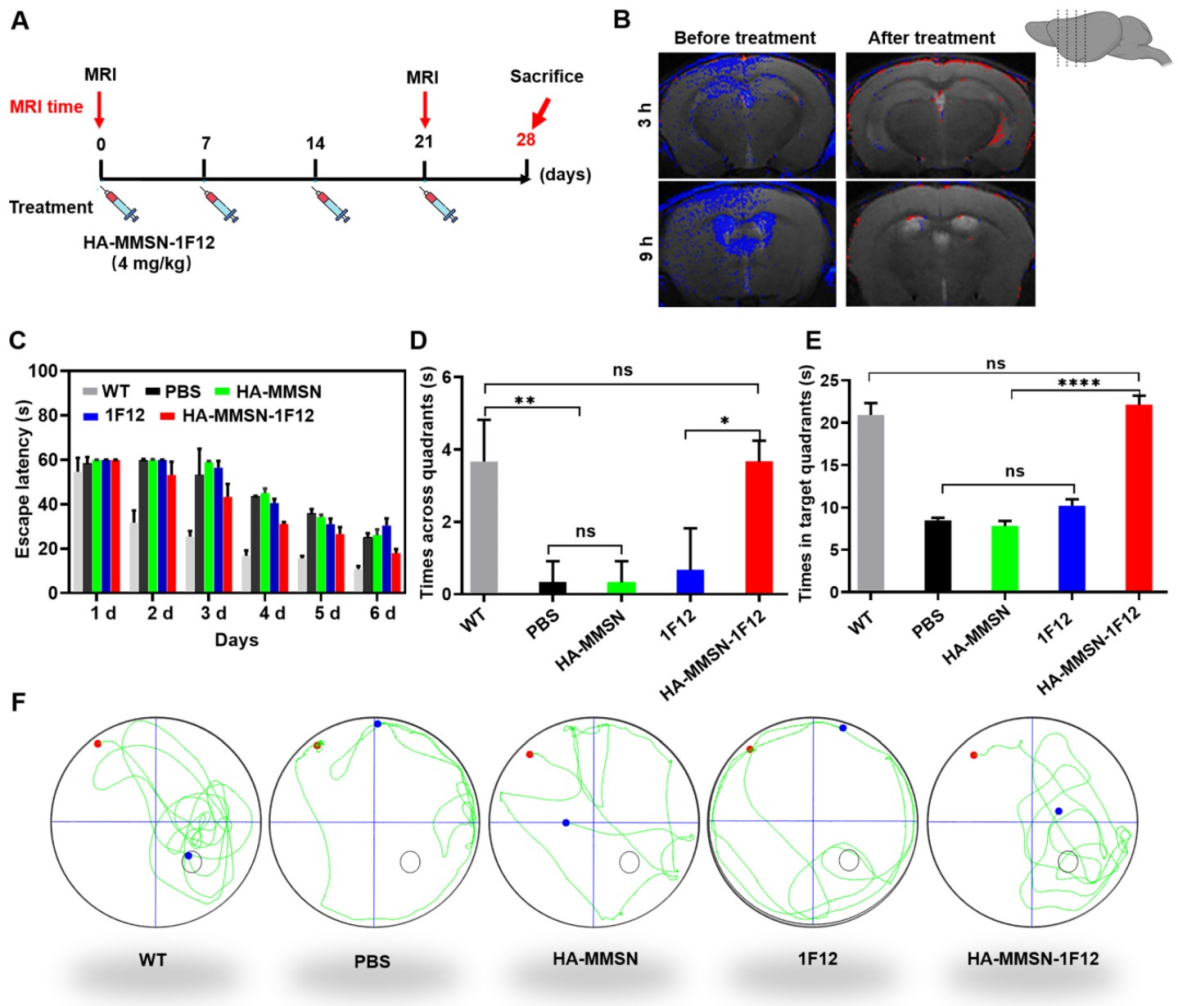
** HA-MMSN-1F12 improves cognitive impairment. (A)** Schematic diagram showing the treatment of ten-month-old APP/PS1 mice through intravenous injection of HA-MMSN-1F12; n = 3 per group. **(B)** Magnetic resonance imaging of the brain 3 and 9 h post-injection of HA-MMAN-1F12 in ten-month-old APP/PS1 mice pretreated with either 4 mg/kg HA-MMAN-1F12 or PBS for three weeks.** (C)** Escape latency to the platform during the training phase. **(D)** Platform entries. **(E)** Time spent within the target quadrant. **(F)** Representative swimming paths of mice within the probe test. Two-way analysis of variance (ANOVA) was performed to undergo multigroup comparisons. Statistical significance is indicated with ^*^p < 0.05, ^**^p < 0.01, ^****^p < 0.0001, and n.s. (no significance).

We performed MRI by intravenous injection of HA-MMSN-1F12 into ten-month-old APP/PS1 mice pretreated with HA-MMSN-1F12 for three weeks to evaluate its therapeutic effect (4 mg/kg/week; Figure 6A). PBS was used as a control, and brain MRI was performed 3 and 9 h after post-injection of HA-MMSN-1F12. Results showed robust T2 MRI signals (blue) in the cortex and hippocampus of APP/PS1 mice. In contrast, T2 MRI signals were weaker in APP/PS1 mice treated with HA-MMSN-1F12 (Figure 6B), indicating that HA-MMSN-1F12 significantly reduced Aβ plaques in the brains of APP/PS1 mice.

AD is a progressive neurodegenerative disease that results in progressive cognitive impairment. We studied whether intravenous administration of HA-MMSN-1F12 could effectively improve cognitive deficits based on MRI results. Next, the Morris water maze test evaluated the effect of HA-MMSN-1F12 on cognitive deficits in APP/PS1 mice. Compared to WT mice, APP/PS1 mice took longer to reach the underwater platform during training trials (Figure 6C) and made fewer crossings inside the target zone of the underwater platform (Figure 6D). In addition, they swam less in the target quadrant on the sixth day (Figure 6E), indicating significant spatial memory deficits. Notably, HA-MMSN and 1F12 treatments failed to statistically improve memory function compared to control APP/PS1 mice (Figure 6D-F). However, t learning and memory abilities were significantly improved after HA-MMSN-1F12 administration (Figure 6D-F). Furthermore, HA-MMSN-1F12-treated APP/PS1 mice exhibited spatially oriented swimming behavior (Figure 6F).

### Assessing the effect of HA-MMSN-1F12 on reducing brain Aβ burden

**Figure 7:**
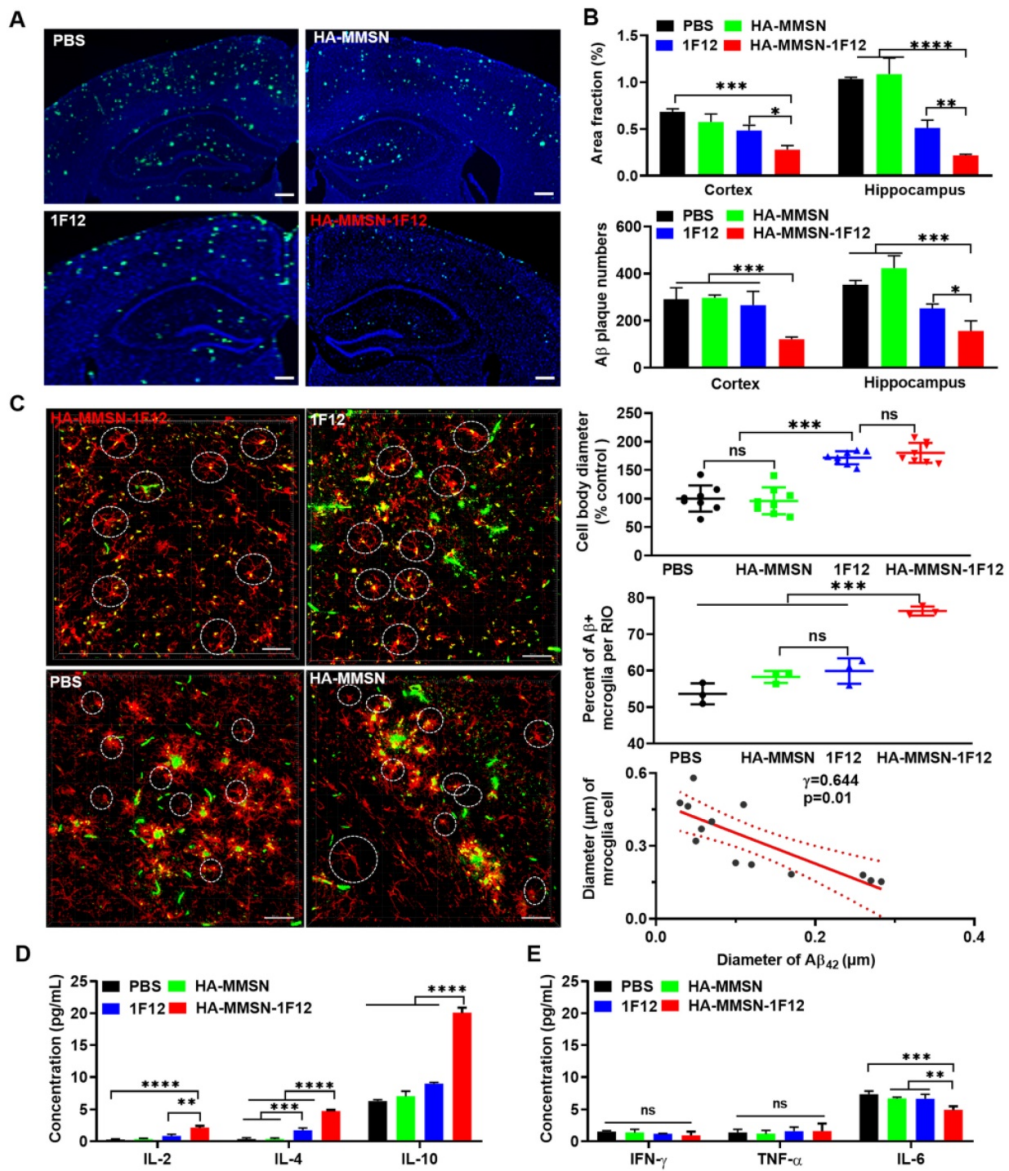
** Intravenous HA-MMSN-1F12 improves the Aβ burden and neuroinflammation in the brain. (A)** Representative images of the brain sections using ThioS staining in ten-month-old APP/PS1 mice after treatment using PBS, HA-MMSN, 1F12, and HA-MMSN-1F12; n = 3 per group. ThioS (green) assessed the number of Aβ plaques after treatment; scale bar, 100 µm. **(B)** Area fraction and number of Aβ plaques in the hippocampus and cortex of ten-month-old APP/PS1 mice treated with PBS, HA-MMSN, 1F12, and HA-MMSN-1F12. **(C)** Representative images of microglia and Aβ plaques after immunostaining using anti-IBA1 and anti-Aβ (6E10) within the hippocampal CA1 region of ten-month-old APP/PS1 mice treated with PBS, HA-MMSN, 1F12, or HA-MMSN-1F12. Quantitative analysis of the diameter of IBA1-positive microglial cell bodies, the percentage of Aβ-positive microglia (white cycle), and the correlation between the diameter changes of IBA1-positive microglial cell bodies and the Aβ plaque diameter (Pearson's correlation) from ten-month-old APP/PS1 mice treated with PBS, HA-MMSN, 1F12, or HA-MMSN-1F12. IBA1 (red), 6E10 (green), and scale bar, 500 µm. Quantitative analysis expressing **(D)** anti-inflammatory factors (IL-2, IL-4, and IL-10) and **(E)** pro-inflammatory factors (IFN-γ, TNF-α, and IL-6). Two-way analysis of variance (ANOVA) was undergone for multigroup comparisons. Statistical significance is considered when ^*^p < 0.05, ^**^p < 0.01, ^***^p < 0.001, ^****^p < 0.0001, and n.s (indicating no significance).

We investigated whether intravenous administration of HA-MMSN-1F12 reduces brain Aβ. HA-MMSN-1F12-treated mice had significantly lower ThioS-positive Aβ plaques in the cortex (*p* < 0.0009) and hippocampus (*p* < 0.0001) than PBS-treated mice (Figure 7A and Figure S12). The number of Aβ plaques in the cortex (*p* = 0.0013) and hippocampus (*p* = 0.0303; Figure 7B) of mice treated with HA-MMSN-1F12 was significantly reduced compared to mice treated with 1F12.

Next, we investigated whether intravenous injection of HA-MMSN-1F12 could inhibit microglial activation. The area fraction of CD86-positive microglia in the cortex and hippocampus was significantly reduced (*p* < 0.0001) in HA-MMSN-1F12- and 1F12-treated mice compared to PBS-treated mice (Figure S13A-B). In addition, the number of CD86-positive microglia in the cortex (*p* = 0.0379) was significantly lower than that in 1F12-treated mice, but not in the hippocampus (*p* = 0.1828) of HA-MMSN-1F12-treated mice. Blockade of microglial activation in AD models has been found to be neuroprotective 34, and massive Aβ accumulation is accompanied by local microglial activation 35. Therefore, the interaction between microglia and Aβ was further investigated. Immunofluorescence staining of the cerebral cortex of PBS-treated mice showed swelling of microglia, branching cells with larger cell bodies and shorter protrusions, and activated microglia surrounded by extensive Aβ plaques (Figure 7C and Figure S14), consistent with previous findings 36, 37. HA-MMSN-1F12-treated mice had more small Aβ plaques and microglia with quiescent branching morphology. 1F12-treated mice exhibited a similar phenomenon, which was not as pronounced as HA-MMSN-1F12-treated mice (Figure 7C). Quantitative analysis revealed that HA-MMSN-1F12-treated mice had the longest microglia body diameter (*p* < 0.0001) and the highest resting cell number (*p* < 0.0001) in the cortex (Figure 7C) than PBS-treated mice. Moreover, regression analysis revealed that the diameter of cell bodies depended on the size of Aβ plaques (Figure 7C). Therefore, these results confirmed that HA-MMSN-1F12 reduced Aβ plaque burden in the brain by depolymerizing and blocking microglial activation.

Next, we investigated whether intravenous administration of HA-MMSN-1F12 could attenuate neuroinflammation. The levels of the anti-inflammatory cytokines interleukin 2 (IL-2) and interleukin 4 (IL-4) were significantly increased in HA-MMSN-1F12-treated mice (*p* < 0.0001; Figure 7D) compared to PBS-treated mice. However, intravenous administration of HA-MMSN-1F12 significantly increased IL-2 (*p* = 0.0016), IL-4 (*p* < 0.0001) and interleukin 10 (IL-10) (*p* < 0.0001) compared to the 1F12-treated mice (Figure 7D). Furthermore, HA-MMSN-1F12 significantly decreased the pro-inflammatory cytokine interleukin 6 (IL-6) compared with PBS (*p* = 0.0002), MMSN (*p* = 0.0053), and 1F12 groups (*p* = 0.0073; Figure 7E). Tumor necrosis factor-α (TNF-α) and interferon-γ (IFN-γ) levels were not significantly affected in all treatment groups. Therefore, intravenously injected HA-MMSN-1F12 significantly increased anti-inflammatory factors and decreased the levels of pro-inflammatory factor IL-6 in the brain.

Then, the underlying mechanisms of reducing brain Aβ burden and attenuating neuroinflammation after intravenous injection of HA-MMSN-1F12 were explored. Transcriptomic analysis of the hippocampus of the three groups identified a series of differentially expressed genes (DEGs) (Figure S15). Among the down-regulated DEGs, HA-MMSN-1F12-treated mice depicted lower expression levels of mitochondrially encoded ATP synthase membrane subunit 6 (mt-Atp6) and subunit 8 (mt-Atp8) than 1F12- and HA-MMSN-treated mice (Figure S15). This suggests that HA-MMSN-1F12 can ameliorate mitochondrial dysfunction in APP/PS1 mice, an early event in AD pathogenesis due to progressive accumulations of mitochondrial Aβ 38. Transcriptome results from the two groups were compared for the unequivocal discovery of variable genes. Transcriptomes of HA-MMSN-1F12- and 1F12-treated mice showed 140 up-regulated and 291 down-regulated genes. In contrast, the transcriptomes of HA-MSN-1F12- and HA-MMSN-treated mice showed 113 up-regulated genes and 165 down-regulated genes (Figure S16A). Hierarchical cluster analysis of DEGs showed that transcriptomic profiles could effectively distinguish samples from the two treatment groups, indicating different expression patterns within the treatment groups (Figure S16 B-C). Next, KEGG pathway analysis was performed on the DEGs between the two groups. Based on enriched gene counts, several important signaling pathways were related to the immune system and its diseases, nervous system, and neurodegenerative diseases (Figure S17A-B). Finally, the DEG-based co-expression network identified a core node gene called tyrosine hydroxylase (Th), which plays an important role in dopamine synthesis (Figure S18). The down-regulated expression of Th directly affects the synthesis and secretion of dopamine. However, the down-regulation of Th gene and its association with AD pathology remains unclear.

### Biosafety evaluation of HA-MMSN-1F12

According to previous reports, the complete degradation of conventional MMSN takes several weeks 39. MMSN was optimized during synthesis, and its porosity was changed. Therefore, we tried to study its biodegradability. HA-MMSN-1F12 was incubated with simulated body fluid (Krebs-Henseleit solution) for 48 h at 37 °C. TEM showed rapid degradation of silica in HA-MMSN-1F12 (Figure S19A), confirming the excellent bio-degradability of MMSN. In addition, HA-MMSN-1F12 was injected into eight-month-old C57BL/6 mice to evaluate the potential toxicity of HA-MMSN-1F12 *in vivo*, and the blood was collected for biochemical analysis. No measurable adverse effects were observed in liver (ALB, 22.33 ± 0.57 g/L; ALP, 143.33 ± 9.01 IU/L; and ALT, 38.00 ± 1.00 IU/L) and renal (BUN, 10.77 ± 0.46 mmol/L; CRE, 84.00 ± 4.36 μmol/L; and GLOB, 32 ± 1.73 g/L) functions in HA-MMSN-1F12-treated mice (Figure S19B). In addition, no significant differences in histology were observed between HA-MMSN-1F12 and PBS controls. Moreover, no obvious pathological abnormalities were observed in the heart, liver, spleen, lung, and kidney (Figure S19C), indicating that HA-MMSN-1F12 has extremely high biosafety.

## Discussion

This study reported a unique Aβ removal strategy based on large-sized multifunctional MMSN with surface-coupled anti-Aβ_42_ mAb 1F12 that captures peripheral soluble Aβ_42_ and the brain CD44 ligand HA for enhanced brain entry efficiency. Intravenous administration of HA-MMSN-1F12 for three weeks significantly reduced brain Aβ burden, neuroinflammation, and cognitive deficits in APP/PS1 mice. HA-MMSN-1F12 could depolymerize insoluble Aβ aggregates into soluble Aβ monomers in the brain and excrete Aβ through the intestinal metabolic pathways.

Studies have observed that CD44 localizes to the lipid valve of cerebral vascular endothelial cells. Specific binding to the ligand HA induces the synthesis of downstream protein kinase C-α (PKC-α). Thus, the reorganization of the cerebrovascular endothelial cytoskeleton and the formation of pseudopodia are triggered to exert their phagocytic function into the brain 40-42. Notably, CD44 was also highly expressed in the brains of APP/PS1 mice (Figure S8). Therefore, the HA/CD44 interaction may be a potential target of the entire BBB for AD therapy. In addition, HA facilitated the passage of its conjugate MMSN-1F12 through vascular endothelial cells via an *in vitro* BBB model (Figure 3A). Furthermore, *in vivo* experiments showed that intravenously injected HA-MMSN-1F12 was present in the brain of APP/PS1 mice up to 1.57 ± 0.06 %ID/g and specifically labeled Aβ_42_ in the brain (Figure 3B-E). Our findings suggest that HA can assist drugs in crossing the BBB and be used to treat neurological disorders. After intravenous administration of HA-coupled MMSN-1F12, brain Aβ levels and neuroinflammation were significantly reduced in APP/PS1 mice, with improved cognitive function (Figures 6 and 7). In addition, HA-MMSN-1F12 was rapidly degraded after 48 h (Figure S19A), suggesting the safety and efficacy of HA-MMSN-1F12 in clearing central Aβ.

The liver sequesters up to 70% of nanomaterials and only 0.7% is delivered to the target 43, resulting in inefficient drug delivery and poor therapeutic efficacy. Studies have depicted a clear correlation between macrophage uptake and efficient targeted delivery 44, 45. The physicochemical properties of nanomaterials, such as size, shape, charge, and "stealth" surface, affect their interaction with macrophages and blood half-life 46, 47. Within a certain size range, the larger the particle size of spherical nanomaterials, the easier they are to be internalized by macrophages 48. In this study, the low enrichment of HA-MMSN-1F12 in the liver was associated with its non-sphericity (Figure 1B-C). In addition, HA is very hydrophilic, so HA-MMSN-1F12 has a high negative charge (-13.03 ± 0.12 mV). It could help HA-MMSN-1F12 avoid interacting with opsonins, thereby reducing the accumulation of macrophages (Figures 3 and 4). The hepatic uptake was low at 3 h after intravenous injection of 4 mg/kg HA-MMSN-1F12, only 0.07-0.50 %ID/g in the liver, indicating that the large particle size of HA-MMSN-1F12 can escape the uptake of hepatic KCs (Figure 3 and 4). Therefore, the characteristic physicochemical properties of HA-MMSN-1F12 can increase the concentration of HA-MMSN-1F12 in peripheral blood and improve its efficiency across the BBB in the brain.

Aβ levels in the central nervous system are 20 to 67 times higher than in the periphery. Aβ levels in the brains AD mice can increase by more than 30% within three months 49. However, clinical results indicate that intravenous infusion of Aβ antibodies has limited effect in reducing Aβ burden in the brain. In the current study, peripheral blood Aβ_42_ levels reached a minimum at 24 h and increased again at 216 h, higher than baseline after a single intravenous injection of HA-MMSN-1F12 (Figure 5B). Blood soluble Aβ_42_ levels were positively correlated with brain Aβ levels. Thus, peripheral blood Aβ_42_ levels were higher than baseline at 216 h, indicating elevated brain soluble Aβ_42_ levels. *In vitro* simulation experiments confirmed that HA-MMSN-1F12 could degrade Aβ_42_ aggregates into soluble Aβ_42_ species (Figure 2). Furthermore, 1F12 and HA-MMSN-1F12 showed no significant difference in reducing Aβ_42_ levels in peripheral blood. The immunofluorescence results of brain sections showed that the Aβ plaques in the brains of mice in the HA-MMSN-1F12 treatment group were smaller than those in the 1F12 treatment group (Figure 7). Targeting the depolymerization of Aβ plaques in the brain and the rapid excretion of peripheral soluble Aβ by intestinal metabolism is characterized by the excellent therapeutic effect of HA-MMSN-1F12 in reducing the Aβ burden in the brain.

The clinical diagnosis of AD includes positron emission tomography (PET) and cerebrospinal fluid (CSF) detection 50, 51. PET is expensive and radioactive, while CSF is invasive. MRI is not invasive and radioactive and less expensive than PET scanning but lacks Aβ-specific MRI probes 52, 53. The development of Aβ-specific MR probes is significant for the diagnosis of AD. The developed HA-MMSN-1F12 showed high binding affinity (Kd = 1.27 ± 0.34 nM) to Aβ_42_Os *in vitro* (Figure 1D). In addition, the HA-MMSN-1F12 probe enables specific MR imaging of Aβ plaques in the brains of APP/PS1 mice to understand the pathological development of AD (Figure 6B), providing an excellent MRI probe value for AD diagnosis. HA-MMSN-1F12 MRI can identify structural information and molecular target locations during early Aβ accumulation. Therefore, HA-MMSN-1F12 has the potential to evaluate drug candidate efficacy and early diagnosis of AD.

As a major player in neuroinflammation, microglia are crucial for the initiation and development of AD 54. Most studies enhance Aβ clearance by activating microglia 55. However, activation of microglia releases pro-inflammatory cytokines that further exacerbate inflammation, which in turn enhances Aβ production and neuroinflammation, leading to neuronal degeneration and necrosis. In this study, when Aβ plaques in the brain were cleared by HA-MMSN-1F12, the number of activated microglia decreased, generating more microglia with quiescent branching morphology (Figure 7), which resulted in the amelioration of cognitive deficits in AD mice (Figure 6). In addition, intravenous administration of HA-MMSN-1F12 increased the production and release of inflammatory cytokines, including IL-2, IL-4, and IL-10, resulting in anti-inflammatory responses (Figure 7D), promoting the improvement of neuroinflammation in AD. Therefore, restoring the physiological functions of microglia to alleviate neuroinflammation is also the key to AD treatment.

Based on the previously developed anti-Aβ_42_ mAb 1F12, we demonstrate that HA-MMSN-1F12, obtained by modifying the MMSN surface with HA and 1F12, reduces brain Aβ burden and neuroinflammation, and improves cognitive deficits in APP/PS1 mice. The superior efficacy of HA-MMSN-1F12 is related to its design, including 400-600 nm non-spherical particles and injectable doses to reduce hepatic clearance and degrade Aβ aggregates in the brain into soluble Aβ_42_ species that are excreted through intestinal metabolism. This study provides a new strategy to overcome off-target nanomaterial accumulation in the liver and excrete disease-causing biohazards through intestinal metabolism, providing a new therapeutic avenue for combating brain diseases.

## Materials and methods

### Chemicals and materials

Aβ_40_, Aβ_42_, and Aβ_42_Arc peptides were lyophilized powders custom-synthesized from Royo Biotech Co., Ltd (Shanghai, Chian), with a purity of > 95%. Anti-Aβ (6E10) and Anti-CD86 antibodies were obtained from Invitrogen (Carlsbad, CA, USA). Anti-Iba1 (019-19741) was acquired from Wako laboratory chemicals (Osaka, Japan). An anti-CD44 antibody was obtained from Sevier Bio (Wuhan, China). Goat anti-mouse IgG (H+L) and protein A resin were ordered from GenScript (Nanjing, China). Pierce streptavidin-coupled Poly-Horseradish Peroxidase (HRP) and protein marker were purchased from Thermo Fisher Scientific. Thioflavin T (ThioT), Thioflavin S (ThioS), Dimethyl sulfoxide (DMSO), and bovine serum albumin (BSA) were ordered from Sigma-Aldrich. N-hydroxysuccinimide (NHS) ester and Cy5-NHS ester were purchased from Lumiprobe (Hannover, Germany). FeCl_2_·4H_2_O was provided by Ourchem (Zhejiang, China). 4',6-diamidino-2-phenylindole (DAPI) was ordered from Beyotime Biotechnology (Shanghai, China). FeCl_3_·6H_2_O was provided by Lingfeng Chemical Reagents (Shanghai, China). 1-(3-Dimethylaminopropyl)-3-ethyl carbodiimide hydro (EDC), NHS (98%), Oleic acid (OA, AR), hyaluronic acid (HA), N-cetyltrimethylammonium bromide (CTAB, 99%), Ethyl acetate (EtOAc, 99.5%) and methanol were purchased from Aladdin (Shanghai, China). Tetraethyl orthosilicate (TEOS, 98%) was obtained from Macklin (Shanghai, China). All other chemicals were purchased from commercial suppliers and used as received.

### Synthesis of magnetic mesoporous silica nanospheres (MMSN)

Firstly, monodisperse superparamagnetic iron oxide nanoparticles coated with oleic acid (Fe_3_O_4_@OA) nanoparticles were prepared by the chemical co-precipitation technique 56. Briefly, 33.5 mmol FeCl_2_·4H_2_O and 41.8 mmol FeCl_3_·6H_2_O were added to 80 mL of deionized water and heated to 80 °C under a nitrogen atmosphere. After vigorous stirring for 0.5 h, 45 mL of ammonium hydroxide and 2 mL of OA were added and reacted for 25 min to obtain Fe_3_O_4_@OA. Secondly, MMSN nanoparticles were synthesized using a sol-gel method with some modifications 57, 58. 7.5 mg of Fe_3_O_4_@OA was dissolved in 500 μL of chloroform and mixed with 0.1 g of CTAB in 5 mL of water. After vigorous stirring, a homogeneous oil-in-water microemulsion was obtained. After evaporating chloroform for 10 min at 60 °C, the resulting water-dispersed nanoparticles were diluted with 100 mL of water. Then, 22.70 mmol/L of ammonium hydroxide, 2.23 mmol/L of TEOS, and 51.10 mmol/L of EtOAc were added to the diluted aqueous solution containing magnetite nanoparticles. The resulting mixture was reacted for 30 s and centrifuged at 80 rpm to obtain MMSN.

### Functional modification of HA-MMSN

The surface of MMSNs was modified with HA-NHS, that is, 10.8 μmol/L HA was dissolved in 500 μL DMSO, and then 10.8 μmol/L EDC and 54 μmol/L NHS were added to the solution to activate HA. After adding activated HA-NHS to the MMSN solution for 3 h, the product was lyophilized to generate the corresponding HA-MMSN-NHS nanoparticles.

Conjugation of antibodies to HA-MMSNs-NHS was performed by using conventional amidation reaction methods. Briefly, 10 nmol/L of the corresponding antibody (1F12 or IgG) was dissolved in 1 M PBS, adjusted to pH 8.5 with 0.1 M Na_2_CO_3_, and then mixed with 20 nmol/L of HA-MMSN-NHS ester to react for 3-4 h at room temperature. Antibody-conjugated MMSNs were separated by a magnet to remove excess free antibodies. As shown in Figure S2C, representative FT-IR spectra of HA-MMSN-1F12 showed characteristic absorptions at 1653.90 and 3426.27 cm^-1^ (Amide Ⅰ, HN-C=O stretching) and 1542.23 cm^-1^ correspond to -N-H (Amide II) bending frequency, indicating that 1F12 has been successfully coupled to the surface of functionalized HA-MMSN-NHS.

### Preparation of fluorescent dye-labeled 1F12 and HA-MMSN-1F12

Non-immune HA-MMSN-1F12 and 1F12 were conjugated to fluorescent dyes (Cy3-NHS or AlexaFluor 488) using the conventional amidation reaction chemistry method described above. Antibodies were stored at -80 °C before labeling with fluorochromes, and fluorochrome-labeled 1F12 were purified using a size-exclusion PD-10 column with 1 M PBS as mobile phase, and fluorochrome-conjugated HA-MMSN-1F12 were separated by a magnet to remove excess free fluorochrome. As shown in Figure S7, the results of fluorescence intensity and spectrum measurements showed that Cy3-labeled HA-MMSN-1F12 or 1F12 had strong Cy3 fluorescence intensity with a fluorescence peak at 561 nm, which indicated that Cy3 was successfully conjugated with HA-MMSN-1F12 or 1F12.

### Preparation of oligomeric and monomeric Aβ

Aβ_40_ monomers (Aβ_40_Ms), Aβ_42_ monomers (Aβ_42_Ms), and Aβ_42_Arc monomers (Aβ_42_ArcMs) were prepared by dissolving lyophilized Aβ_40_, Aβ_42_, and Aβ_42_Arc peptides in 10 mmol/L NaOH and diluted in 2 M phosphate-buffer saline. The prepared Aβ_40_, Aβ_42_, and Aβ_42_Arc monomers solutions (50 μM) were stored at -20 °C as stock solutions, respectively. Aβ_42_ oligomers (Aβ_42_Os) were obtained by dissolving lyophilized Aβ_42_ peptide in 10 mmol/L NaOH and diluted to 50 μM in 10 M phosphate-buffer saline overnight at 37 °C in the dark. Aβ_42_Arc oligomers solution (Aβ_42_ArcOs) was prepared by incubating its monomer for 1 h at 37 °C in the dark, followed by centrifugation at 17,900 × g for 5 min.

### Cryo-transmission electron microscopy

The patterns of the prepared Aβ_42_Ms, Aβ_42_ArcMs, Aβ_42_Os or Aβ_42_ArcOs, HA-MMSN, and HA-MMSN-1F12 were confirmed by cryo-transmission electron microscopy (Cryo-TEM). Briefly, 5 μL of samples were dropped onto a copper grid, and excess liquid was blotted with filter paper, leaving a thin film of solution on the mesh. The morphology of the above samples was characterized using a Tecnai G20 transmission electron microscope (FEI Ltd, USA). The instrument works in 80 kV and zero-loss bright field mode, showing as much detail as possible and enhancing image contrast at a focus point of 1-2 μm.

### Characterization of HA-MMSN-1F12

Briefly, 1 mg/mL HA-MMSN and HA-MMSN-1F12 were prepared. Dynamic light scattering (DLS; photon correlation spectroscopy) and the Zetasizer Nano-ZS90 system (Malvern Instruments, Worcestershire, UK) were used to measure the size distribution and polydispersity index (PDI) of the nanoparticles.

### Immunoprecipitation and Western blotting

Frozen tissues were homogenized in liquid nitrogen and extracted with tris-buffered saline (TBS, 20 mmol/L tris and 137 mmol/L NaCl, pH 7.6) solution as previously described 55, 59. The homogenized supernatant was centrifuged at 10,000 x g for 30 min at 4 °C to obtain TBS-soluble protein. The supernatant was aliquoted and stored at -80 °C before analysis. According to the manufacturer's guidance, brain homogenate or blood was incubated with 40 μg/mL 1F12-conjugated protein A/G magnetic beads for 30 min at room temperature. Immunoprecipitated proteins were eluted with 0.1 M glycine (pH 3.0) and immediately neutralized to pH 7.4 with neutralization buffer (1 M Tris-HCl, pH 8.5). After boiling for 10 min, the denatured samples in the loading buffer (Boster Biotech, USA) were loaded on a 12% tris-tricine SDS-polyacrylamide gel electrophoresis (SDS-PAGE). Proteins were transferred to polyvinylidene fluoride (PVDF) membranes at 160 mA, 4 °C for 1.5 h, blocked with 5% skimmed milk in PBS-T, and then incubated with primary antibody 1F12 (1: 1,000, 5% skimmed milk) for 2 h at 37 °C. Membranes were washed in PBS-T followed by 1 h incubation with a second antibody HRP-conjugated goat anti-mouse IgG (H+L) (1:4,000, PBS-T). The signal was detected with the ECL substrate (Vazyme, China) on a Tanon 5200 Multi (Shanghai, China).

### Serum protein adsorption of HA-MMSN-1F12

To test whether HA-MMSN-1F12 has adsorption to serum protein, 15% fetal calf serum (FCS; 1 mg/mL) was incubated with HA-MMSN-1F12 at 37 °C for 24 h. Particles were isolated by centrifugation and washed twice with PBS. The particles were then dispersed in SDS buffer solution (10 mg/mL), mixed with Laemmli buffer after sonication for 30 min, and treated at 95 °C for 10 min before SDS-PAGE.

### Sandwich enzyme‑linked immunosorbent assay (ELISA)

Wells of a 96-well EIA/RIA plate were coated with 1 μg/well of 1F12 and diluted in citrate-buffered saline (CBS) overnight at 4 °C. All wells were blocked with 5% skimmed milk in PBS-T buffer for 2 h at 37 °C. Next, standard series of Aβ_40_Ms, Aβ_42_Ms, Aβ_42_ArcMs, Aβ_42_Os, Aβ_42_ArcOs, blood, and organ lysates were added to the wells in triplicate and incubated at 37 °C for 2 h. After washing 5 times with PBS-T, the wells were incubated with 1F12-HRP (1:500) at 37 °C for 1 h, followed by adding 100 μL of soluble TMB substrate solution at 37 °C for 15 min to reveal the immunoreactivity. Finally, plates were analyzed at 450 nm using an Epoch Microplate Spectrophotometer (Bio Tek, USA).

### Indirect ELISA for detecting the specificity of HA-MMSN-1F12

Wells of a 96-well plate (Corning Inc, USA) were coated with 0.5 μg/well of Aβ_40_Ms, Aβ_42_Ms, Aβ_42_ArcMs, Aβ_42_Os, and Aβ_42_ArcOs in citrate-buffered saline (CBS) overnight at 4 °C. Wells coated with PBS were used as negative controls. All wells were blocked with 5% skim milk dissolved in PBS-T (PBS containing 0.1% Tween-20) buffer for 2 h at 37 °C. Next, standard series of HA-MMSN, 1F12, and HA-MMSN-1F12 were added to the wells for 2 h incubation at 37 °C, followed by the addition of a second antibody HRP-conjugated goat anti-mouse IgG (H+L) (1: 4,000, PBS-T) for 1 h incubation at 37 °C. After 5 washes with PBS-T, 100 μL of soluble 3, 3′, 5, 5′-tetramethylbenzidine (TMB) substrate solution (Tiangen Biotech, China) was added for visualization of immunoreactivity. Finally, the plate was analyzed at 450 nm with an Epoch Microplate Spectrophotometer (Bio Tek, USA).

### Indirect ELISA for detecting the binding affinity of HA-MMSN-1F12

Wells of a 96-well plate were coated with 0.5 μg/well of Aβ_40_Ms, Aβ_42_Ms, Aβ_42_ArcMs, Aβ_42_Os, and Aβ_42_ArcOs, and blocked with 5% skimmed milk. Serial dilutions of 1F12, HA-MMSN, and HA-MMSN-1F12 from 1.0 x 10^-5^ mg/mL to 2.5 x 10^-3^ mg/mL were added to each well for 2 h incubation at 37 °C. After incubation with 1F12-HRP (1:500) at 37 °C for 1 h, TMB substrate solution was added to visualize the immunoreactivity.

### Construction of *in vitro* Aβ_42_ aggregate degradation system

To evaluate the ability of HA-MMSN-1F12 to depolymerize insoluble Aβ_42_ plaques, an *in vitro* degradation system of Aβ_42_ aggregates was constructed based on the transwell system. In this system, the upper and lower chambers are separated by a 5 kDa dialysis membrane. 500 μL of 50 μM Aβ_42_ aggregates in the upper chamber were incubated with 1 μM HA-MMSN-1F12 or HA-MMSN-IgG in the lower chamber at 37 °C and the HA-MMSN-1F12 or HA-MMSN-IgG solution in the lower chamber was changed every 3 h. Then before incubation and 1, 3, 6, 9, 12, 15, and 18 h after incubation, 50 μL of the upper chamber solution was taken for ThT fluorescence detection and sandwich ELISA to quantitatively analyze the content of Aβ_42_ aggregate in the upper chamber. TEM was used to detect the morphological changes of Aβ_42_ aggregate degraded into monomers in the upper chamber solution.

### ThT fluorescence assay

The Aβ_42_ aggregate solution obtained from the above Aβ_42_ aggregate degradation assay was added to 100 μL of 15 μM ThT solution, respectively, and incubated in the dark for 15 min. Then, the fluorescence signal was recorded at an emission wavelength of 490 nm (excitation at 440 nm) by using a multiscan FC microplate photometer (Thermos, USA). All fluorescence experiments were repeated three times and averaged together with the standard deviation.

### Aβ_42_ aggregate self-assembly inhibition assay

To evaluate the inhibitory effect of HA-MMSN-1F12 on Aβ_42_ aggregate assembly multimerization, 1 mL of 50 μM Aβ_42_ monomer solution was incubated with 1 μM HA-MMSN-1F12 at 37 °C on a shaker for 0, 1, 3, 6, 9, 12, 15, and 18 h, respectively. 100 μL of the mixed solution was taken at the above different time points for ThT fluorescence detection.

### Construction of the *in vitro* BBB model

To explore whether HA-MMSN-1F12 can cross the BBB and enter neuronal cells, a Transwell model was constructed to simulate the BBB. An *in vitro* BBB model was constructed based on human umbilical vein endothelial cells (HUVECs) for 7 days, and SY5Y cells were inoculated in Transwell lower chambers for 48 h, 1 µM Cy3-labeled 1F12, HA-MMSN-1F12, HA-MMSN, and MMSN was added to the upper chambers at 37 °C. At 24 h, SY5Y cells were observed for confocal imaging.

### Animals

All procedures involving animal studies have been reviewed and approved by the Institutional Animal Care and Use Committee of Huazhong University of Science and Technology. APPSwe/PS1dE9 (APP/PS1) transgenic mice were purchased from Beijing Huafukang Biotechnology Co., Ltd. In this study, APP/PS1 aged 6-10 months was used. All mice were housed in groups (5 mice per cage) under standard specific pathogen-free (SPF) conditions (12 h light/dark cycle, standard temperature 24 °C, humidity 50-60%, and pathogen-free).

### *In vitro* fluorescence imaging and biodistribution of HA-MMSN-1F12

To evaluate the biodistribution of HA-MMSN-1F12, male APP/PS1 mice were divided into 5 groups of eight mice each. Animals were sacrificed at 6 h after intravenous injection of Cy5-labeled MMSN, HA-MMSN, 1F12, and HA-MMSN-1F12. An equal volume of PBS administered intravenously was used as a control. Mice were anesthetized with 0.4 mL Avertin (25 mg/mL) and perfused with 1 M PBS for 30 min. Major organs (brain and intestine) were collected, washed with PBS, weighed, and imaged on a black plate.

The total uptake of Cy5-labeled HA-MMSN-1F12 or other probes in major organs was determined by previously described quantitative methods 60, 61. Here, 50 mg of each organ or blood was extracted by ultra-low temperature grinding, dissolved in 500 μL of 3% (v/v) methanol, and centrifuged at 5,000 g for 10 min at 4 °C to remove debris. Then, 100 μL of supernatant was transferred to a 96-well plate. The plate was analyzed at 647 nm with an Epoch Microplate Spectrophotometer (Bio Tek, USA). Standard curves for quantifying total 1F12 and HA-MMSN in organs were y = 0.007x + 0.044 (R^2^ = 0.9992) and y = 1.0363x + 0.0428 (R^2^ = 0.9994), respectively, x stands for 1F12 or HA-MMSN concentration, and y stands for fluorescence (Cy5) absorbance value. Under these conditions, the equation for %ID/g is [(C_organ_ × V_organ_) / g_organ_] / ID/g × 100, V_organ_ (mL) represents the tissue grind extraction volume of the region of interest, C_organ_ (mg/mL) represents the concentration of 1F12 or HA-MMSN in V_organ_ calculated according to the above standard curve, g_organ_ (g) represents the tissue weight of the region of interest, ID/g represents injected dose (4 mg/kg) in mice.

### Immunofluorescence assays of slices

APP/PS1 mice were anesthetized with 0.4 mL Avertin (25 mg/mL) and perfused transcardially with 4% paraformaldehyde (PFA) for 30 min. The brain tissues were immersed in 4% PFA and stored in 30% sucrose in PBS. Tissues were sliced to a thickness of 25 μm using a frozen Leica CM3050S microtome and mounted on an adhesion microscope slide (Jiangsu, China). Each tissue section was washed 3 times with 1 M PBS for 10 min and then washed with 0.2% Triton X-100 (Macklin, China) at room temperature for 20 min. The sections were blocked with 3% BSA for 2 h at room temperature and incubated with Cy5-labeled HA-MMSN-1F12, 1F12, or HA-MMSN-IgG overnight at 4 °C. Finally, all slices were washed with 1 M PBS and stained with ThioS. The Zeiss LSM710 microscope was used to image slices with Cy5, Alexa Fluor 488, and DAPI filters.

### Thioflavin S counterstain

Aβ was counterstained with ThioS as previously described 62. APP/PS1 tissues were sectioned 25 μm thick and immunolabeled with Cy5-labeled HA-MMSN-1F12 or 1F12. Sections with or without Cy5 labeling were incubated with 0.002% ThioS solution in TBS (diluted from 0.02% ThioS stock solution in distilled water) for 30 min, then washed 3 times in 50% ethanol (v/v), with a final wash in 1 M PBS 3 times for 5 min each. Sections were mounted with 50% glycerol reagent (m/v) and images were acquired under fluorescence microscopy at 10x magnification and analyzed by Image J software.

### Flow cytometric analysis of KCs

Flow cytometric analysis of KCs was performed using previously described methods 63, 64. The hepatic low-pressure portal vein was perfused with collagenase type IV solution and digested with collagenase type IV and DNase I at 37 °C. The mixture was filtered using a 70 μm cell strainer (BD, USA), and tissue debris was removed by centrifugation at 400 × g for 2 min to obtain hepatocytes. The cell pellets were resuspended in 3 mL of RPMI1640 medium, and 5 mL of 70% Percoll and 7 mL of 30% Percoll were added to the cell solution along the wall of the 15 mL tube. KCs were obtained after centrifugation at 500 × g for 20 min to remove the intermediate layer.

KCs (1×10^5^/well) were incubated with Alexa Fluor 488-labeled 1 μM HA-MMSN-1F12, HA-MMSN, MMSN, and 1F12 for 2 h at 37 °C in a humidified incubator with 5% CO_2_ to analyze the phagocytosis of nanoparticles by KCs. Then, KCs were washed with PBS and stained with AlexaFluor 647-labeled anti-mouse F4/80 (1:400, Abcam, England). After 1 h incubation, KCs were washed three times with PBS to remove excess AlexaFluor 647-labeled anti-mouse F4/80. Finally, the fluorescence intensity of KCs was examined using a CytoFLEX flow cytometer (Beckman Coulter, USA). Confocal images of KCs were acquired using a Zeiss LSM710 confocal imaging system (Oberkochen, Germany). Data were analyzed using FlowJo software (FlowJo, Ashland, OR, USA) and ImageJ software, respectively.

### Evaluation of the effect of HA-MMSN-1F12 targeting Aβ in the brain of APP/PS1 mice

To assess the effect of HA-MMSN-1F12 targeting Aβ in the brains of APP/PS1 mice, ten-month-old male APP/PS1 mice (n = 3) were intravenously injected with Cy5-labeled HA-MMSN-1F12 at a dose of 12 mg/kg. At 6 h post-injection, mice were anesthetized with 0.4 mL of Avertin (25 mg/mL) and perfused with 1 M PBS for 30 min. Next, brains were collected, fixed in 4% PFA solution, and completely dehydrated in 30% sucrose solution. Brain tissues were sliced to a thickness of 25 μm using a freezing Leica CM3050S microtome. Finally, all slices were washed with 1 M PBS and stained with ThioS.

### *In vivo* brain MRI of APP/PS1 mice

After injection of 100 μg HA-MMSN-1F12 into APP/PS1 mice, APP/PS1 mouse brains were performed magnetic resonance imaging (MRI) at 0, 3, and 9 h using a Bruker Biospec system (BioSpec 70/20USR, Billerica, MA) at 7.0 T (proton resonance frequency 300M Hz, aperture 20 cm). During the scan, the mice were anesthetized by inhaling 1-1.5% isoflurane in 30%/70% O_2_/N_2_. T2* weighting parameters are as follows: repetition time (TR) = 2500 ms, echo time (ET) = 29 ms, rarity factor = 8, field of view (FOV) = 1.80 cm × 1.80 cm, in-plane resolution = 0.07 cm, single layer thickness = 0.6 cm, a total of 25 slices. T2-weighted images provide more localization of HA-MMSN-1F12 because iron causes local changes in susceptibility to which T2-weighted images are sensitive.

### Immunohistochemistry (IHC)

To detect the expression changes of CD86 in the brain after treatment, the brain tissue of the treated mice was sliced into a thickness of 25 μm. Antigen retrieval buffer at pH 9.0 was used for each brain section at 98 °C for 20 min. Sections were blocked with 3% BSA for 2 h at room temperature and incubated with anti-CD86 primary antibody overnight at 4 °C. All sections were then washed three times with TBS buffer and incubated with alkaline phosphatase (AP) polymeric anti-mouse IgG for 1 h at 37 °C. Finally, the sections were observed under light microscopy (Nikon, Japan).

### Quantitative assays of inflammatory factors

The BD cytometric bead array (CBA) Mouse Th1/Th2/Th17 cytokine kit was used to quantitatively estimate the expression of inflammatory factors in the brain. The procedure was performed according to the protocol provided by the manufacturer (Catalog No.560485, BD).

### Transcriptome analysis

Mouse brain tissue was collected and stored at -80 °C for sequencing. Raw transcriptome data were generated by Illumina's high-throughput sequencing platform. The “Limma” package was used for differentially expressed genes (DEGs) analysis. An empirical Bayesian approach was used to estimate the fold change between cluster 1 and cluster 2, which was determined by a consensus clustering method using a modest t-test 65. The Benjamini-Hochberg correction was used to calculate the adjusted P-value for multiple tests. Genes with an absolute log2 fold change greater than 1.5 were identified as DEGs between the two clusters. DEGs was used as input for pathway analysis. |FC| > 2 and adj.p.val < 0.05 were used as cut-off thresholds. Gene Ontology (GO) and Kyoto Encyclopedia of Genes and Genomes (KEGG) enrichment analyses were performed by the “cluster profile” R package.

### Morris water maze assay

The morris water maze setup consists of a circular tube (diameter, 120 cm; height, 50 cm) and a platform (diameter, 10 cm) that sits 1 cm below the water surface. The standard water temperature was 20-22 °C, and each mouse was tested in 4 quadrants per day for 5 consecutive days. If the mouse finds an underwater platform within 60 s, let it stay on the platform for 30 s. If the mouse does not find an underwater platform within 60 s and stays on the underwater platform for 3 s, it is directed to the platform and stays on the platform for 30 s. On the sixth day, the underwater platform was dismantled. Each mouse was placed in the bathtub from the opposite position to the original quadrant and swim for 60 s.

### *In vitro* biostability of HA-MMSN-1F12

*In vitro* biodegradation of HA-MMSN-1F12 was performed as previously described 58. Briefly, 50 μg of HA-MMSN-1F12 was incubated in Krebs-Henseleit solution (D-glucose 2.0 g/L, magnesium sulfate 0.141 g/L, potassium phosphate monobasic 0.16 g/L, potassium chloride 0.35 g/L, sodium chloride 6.9 g/L, calcium chloride dihydrate 0.373 g/L, and sodium bicarbonate 2.1 g/L) at 37 °C for 12, 24, 48, 72, 96, and 144 h. TEM images of the sample were acquired at the indicated time points.

### Blood biochemical analysis

To evaluate the side effects of HA-MMSN-1F12, 8-week-old male C57BL/6 mice were injected intravenously with 100 mg/kg of HA-MMSN-1F12 per mouse. PBS was used as a control. Blood samples were collected from mice at 24 h after intravenous injection of HA-MMSN-1F12 or PBS. Biochemical analysis was performed using an automated biochemical analyzer (Spotchem EZ SP-4430, Arkray Inc., Kyoto, Japan) and SPOTCHEM II reagent test strips. We performed metabolic measurements in blood, including blood urea nitrogen (BUN), albumin (ALB), alkaline phosphatase (ALP), alanine aminotransferase (ALT), creatinine (CRE), and globulins (GLOB).

### Histopathological analysis

Hearts, livers, spleens, kidneys, and lungs were extracted from mice on day 7 after intravenous injection of HA-MMSN-1F12 or PBS and fixed in 4% PFA solution overnight. Organs were embedded in paraffin, sectioned, and stained with hematoxylin and eosin (H&E). H&E sections were imaged on a Nikon Ni-E microscope (Nikon, Minato, Tokyo, Japan). All images were acquired using NIS-Elements software and further analyzed using ImageJ software.

## Statistical analysis

Statistical analysis was performed using GraphPad Prism version 8.0. All values in the graphs are expressed as mean ± standard error of the mean (SEM) and are used to represent approximately normally distributed continuous variables. One-way or two-way analysis of variance (ANOVA) was used for multiple group comparisons. Statistical significance is indicated in the graph with ^*^p < 0.05, ^**^p < 0.01, ^***^p < 0.001, ^****^p < 0.0001, and n.s. (indicating no significance).

## Supplementary Material

Supplementary figures.

## Abbreviations

Aβ: amyloid-β
AD: Alzheimer's disease
HA: hyaluronic acid
APP: amyloid precursor protein
BBB: blood-brain barrier
mAb: anti-Aβ monoclonal antibody
TfR: Transferrin receptor
LRP1: low-density lipoprotein receptor-related protein-1
FT-IR: Fourier transform infrared
SR: saturation recovery
MSN: Mesoporous silica nanoparticle
MMSN: magnetic mesoporous silica nanosphere
KCs: Kuffer cells
IHC: Immunohistochemistry
MRI: magnetic resonance imaging
HRP: Poly-Horseradish Peroxidase
DMSO: Dimethyl sulfoxide
BSA: bovine serum albumin
NHS: N-hydroxysuccinimide
DAPI: 4',6-diamidino-2-phenylindole
OA: Oleic acid
CTAB: N-cetyltrimethylammonium bromide
TEOS: Tetraethyl orthosilicate
Aβ_40_Ms: Aβ_40_ monomers
Aβ_42_Ms: Aβ_42_ monomers
Aβ_42_ArcMs: Aβ_42_Arc monomers
Aβ_42_ArcO: Aβ_42_Arc oligomer
PBS: phosphate-buffered saline
CBS: citrate-buffered saline
TMB: 3, 3′, 5, 5′-tetramethylbenzidine
TBS: tris-buffered saline
GO: Gene ontology
KEGG: Kyoto encyclopedia of genes and genomes
SDS-PAGE: sulfate-polyacrylamide gel electrophoresis
ThioS: Thioflavin S
DEGs: differentially expressed genes
mt-Atp6: membrane subunit 6
H&E: hematoxylin and eosin
CBA: cytometric bead array
AP: alkaline phosphatase
ET: echo time
FOV: field of view
PFA: paraformaldehyde
SPF: specific pathogen-free
FCS: fetal calf serum
HUVECs: human umbilical vein endothelial cells
PDI: polydispersity index
CSF: cerebrospinal fluid
PKC-α: protein kinase C-α
Th: tyrosine hydroxylase
ALB: albumin
ALP: alkaline phosphatase
ALT: alanine aminotransferase
BUN: blood urea nitrogen
CRE: creatinine
GLOB: globulins
