# Supplementary material for: Dual-targeted magnetic mesoporous silica nanoparticles reduce brain amyloid-β burden via depolymerization and intestinal metabolism
Source: Theranostics. 2022 Sep 11;12(15):6646–64. doi: 10.7150/thno.76574 (PMC9516238; doi:10.7150/thno.76574)
Supplement: Supplementary file 1 — Supplementary figures. [file thnov12p6646s1.pdf]

Supporting Information for

Title: Dual-targeted magnetic mesoporous silica nanoparticles reduce brain amyloid-β burden via depolymerization and intestinal metabolism

Ni. Liu[1,2], Xiaohan Liang[1,2], Changwen Yang[1,2], Shun Hu[1,2], Qingming Luo[1,2,3], Haiming. Luo[1,2]*

[1]Britton Chance Center for Biomedical Photonics, Wuhan National Laboratory for Optoelectronics, Huazhong University of Science and Technology, Wuhan, China

[2]MoE Key Laboratory for Biomedical Photonics, School of Engineering Sciences, Huazhong University of Science and Technology, Wuhan, China

[3]School of Biomedical Engineering, Hainan University, Haikou, Hainan 570228, China

***Correspondence:** Haiming Luo, hemluo@hust.edu.cn；

Britton Chance Center for Biomedical Photonics, Wuhan National Laboratory for Optoelectronics–Huazhong University of Science and Technology, 430074, Wuhan, Hubei, China. Fax: +86-27-87792034; Tel: +86-27-87792033;

Supplementary Figures

Supplementary Figure 1

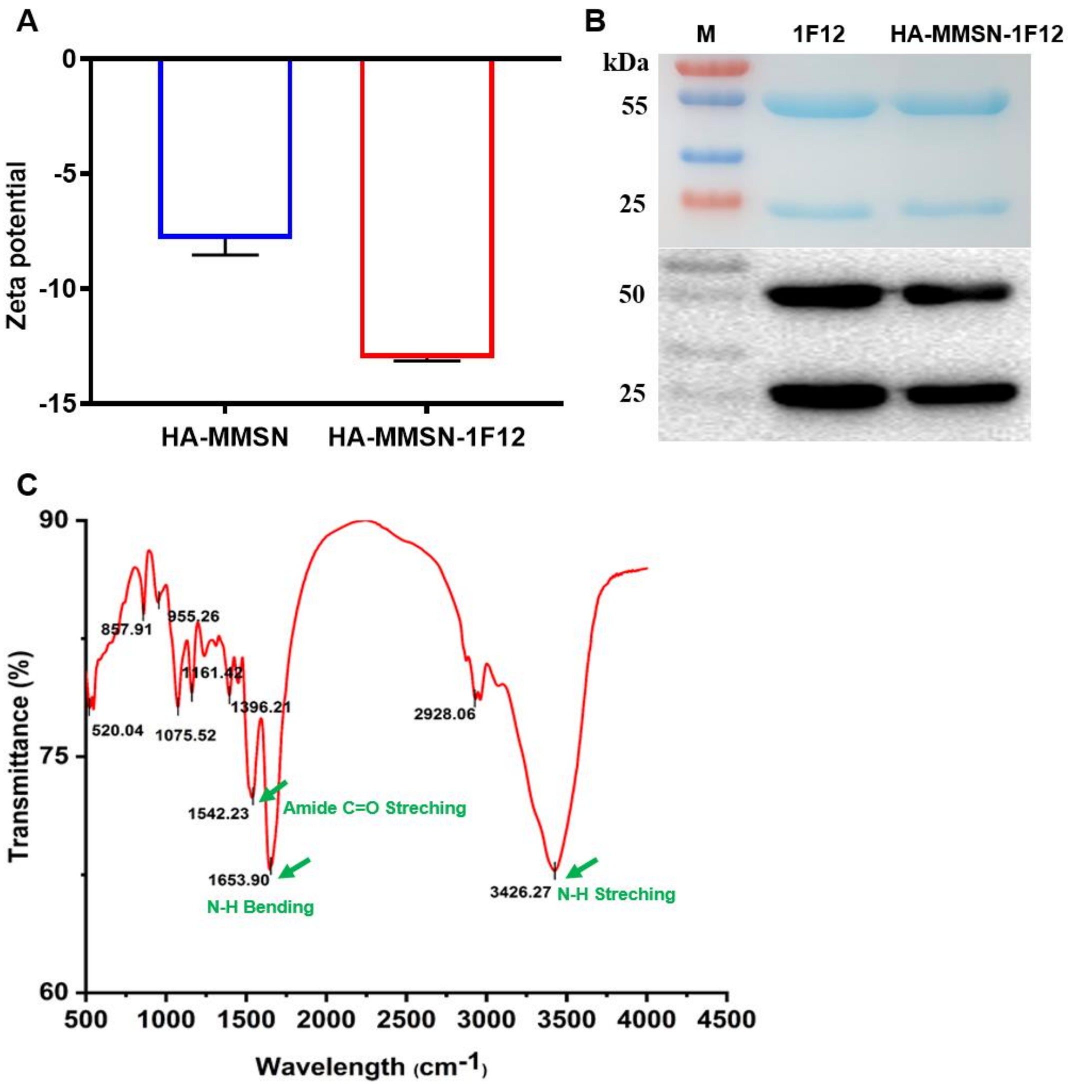

Figure S1. Characterization of HA-MMSN-1F12. (A) Zeta potential of HA-MMSN and HA-MMSN-1F12. **(B)** Successful conjugation of HA-MMSN to 1F12 was assessed by SDS-PAGE and Western blotting. **(C)** FT-IR spectrum of HA-MMSN-1F12.

Supplementary Figure 2

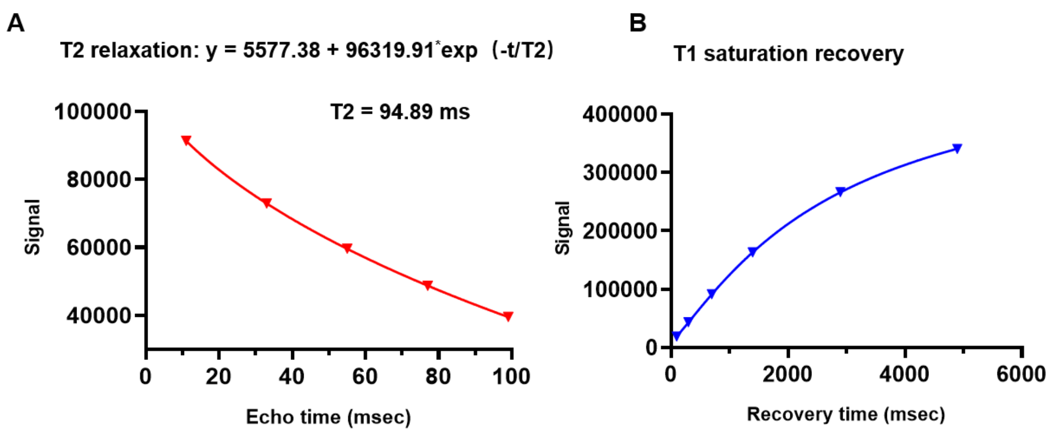

Figure S2. Relaxivity measurements of MMSN. (A) Time courses of the magnetization for T2 relaxation and **(B)** T1 saturation recovery.

Supplementary Figure 3

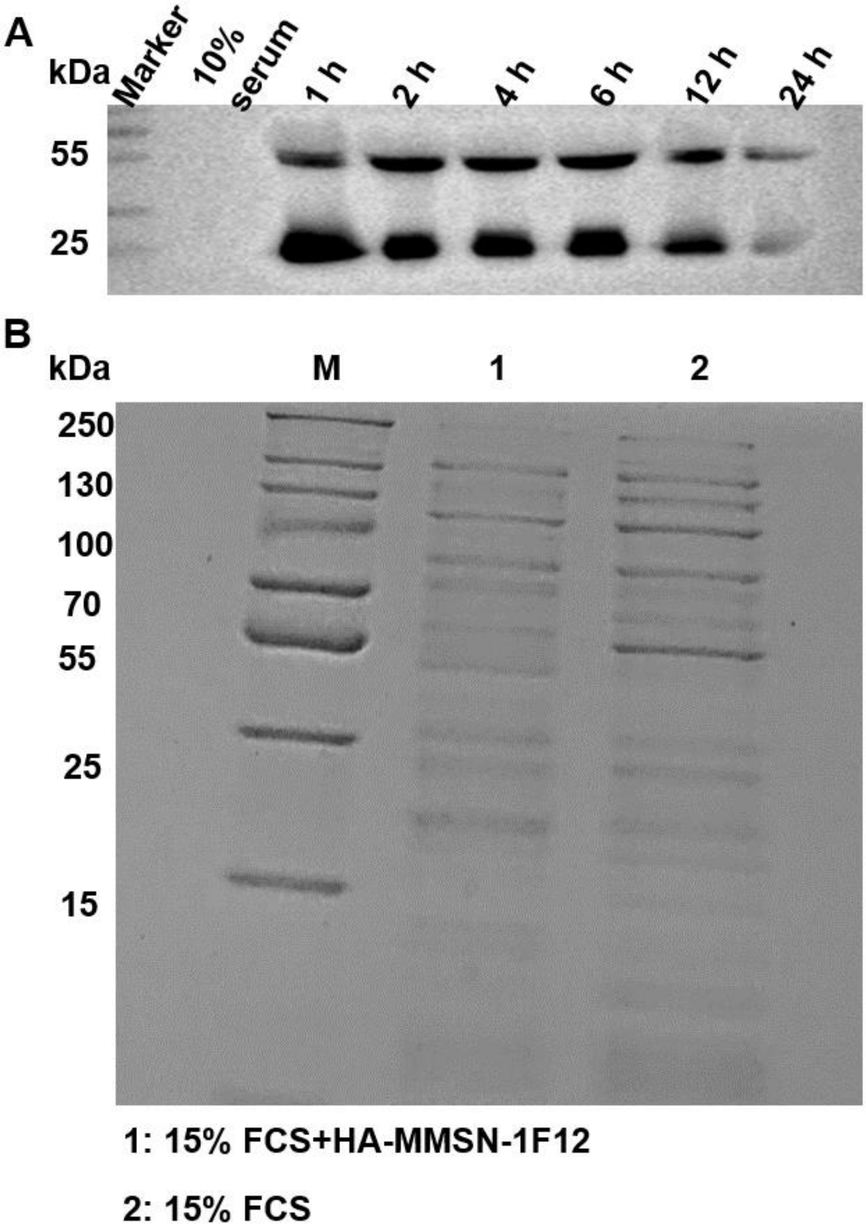

Figure S3. Stability and protein adsorption of HA-MMSN-1F12 in serum. (A) Stability of HA-MMSN-1F12 incubated with 10% mouse serum based on SDS-PAGE. **(B)** The composition of adsorbed proteins on HA-MMSN-1F12 particles was analyzed with SDS-PAGE.

Supplementary Figure 4

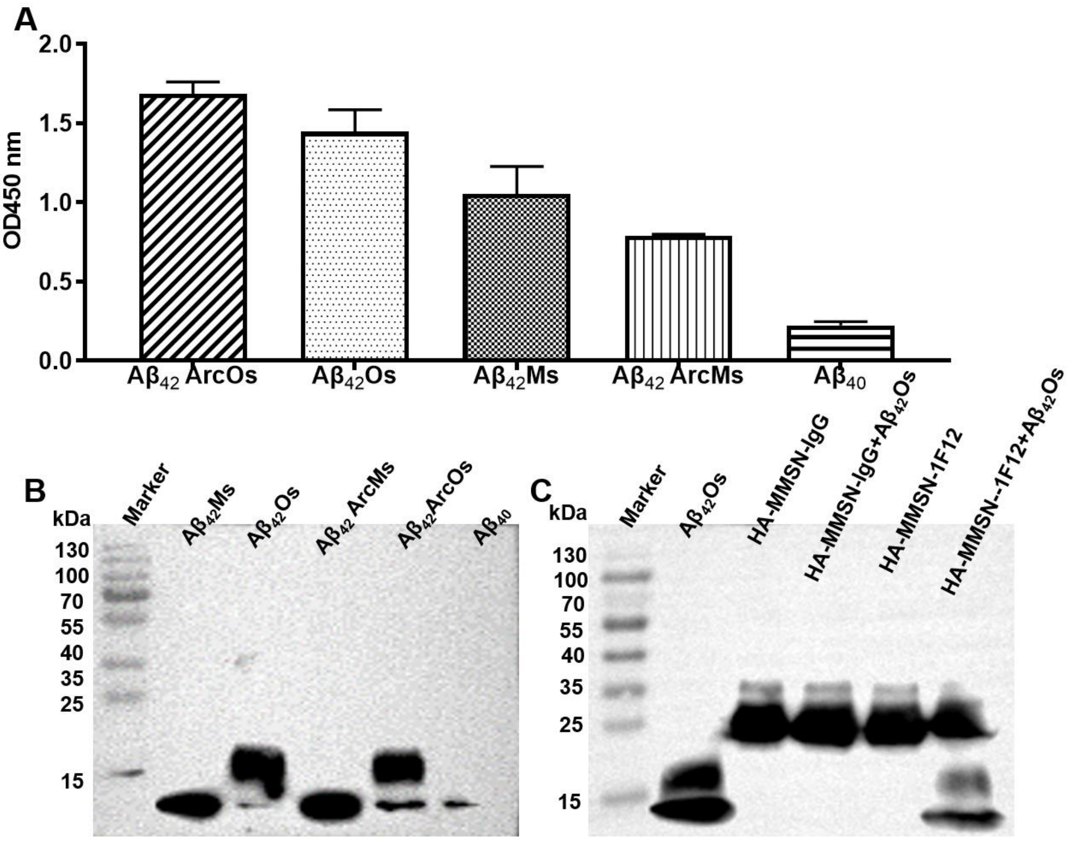

Figure S4. Specificity and affinity of HA-MMSN-1F12. (A) ELISA showed that HA-MMSN-1F12 maintained the binding affinity of 1F12 toward Aβ_{42} species (n = 3). **(B)** Western blotting was used to determine the binding specificity of HA-MMSN-1F12 to different Aβ_{42} species. **(C)** IP-Western blotting of HA-MMNS-IgG and HA-MMSN-1F12 incubated with or without Aβ_{42}Os to show the specificity of HA-MMSN-1F12 binding to Aβ_{42}Os.

Supplementary Figure 5

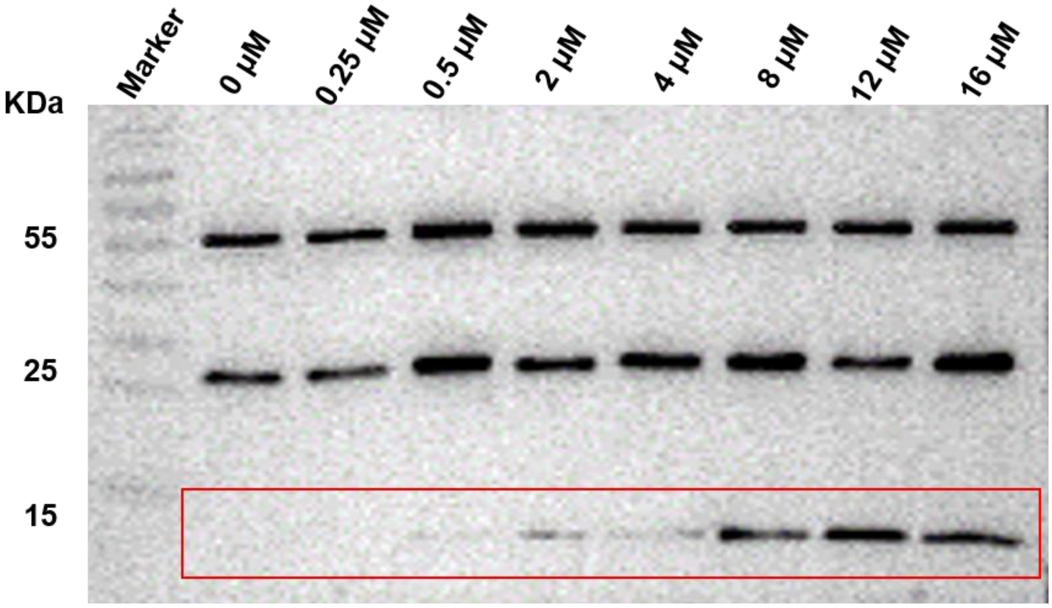

Figure S5. The detection limit of HA-MMSN-1F12 for $A\beta_{42}$Os was evaluated based on Western blotting after incubation of HA-MMSN-1F12 with a series of different concentrations of $A\beta_{42}$Os.

Supplementary Figure 6

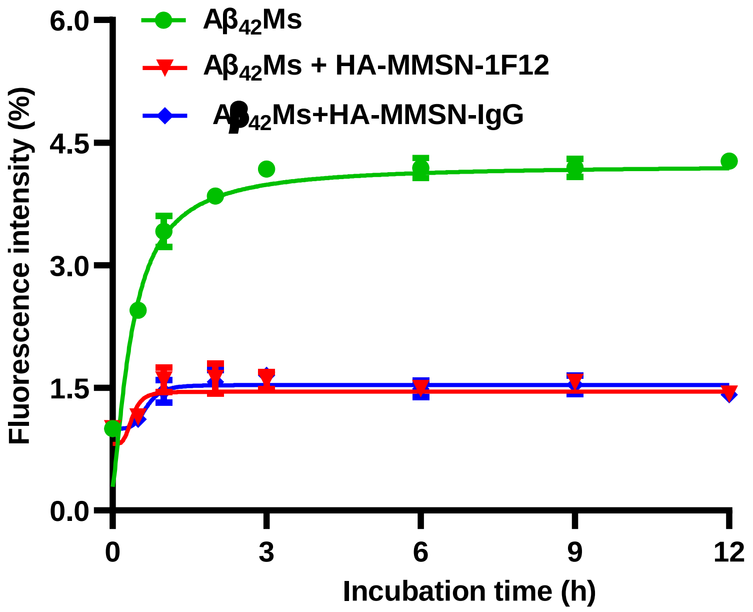

Figure S6. Changes in the levels of Aβ$_{42}$Os during HA-MMSN-1F12 or HA-MMSN-IgG inhibition of Aβ$_{42}$Ms fibrosis were monitored by ThT fluorescence.

Supplementary Figure 7

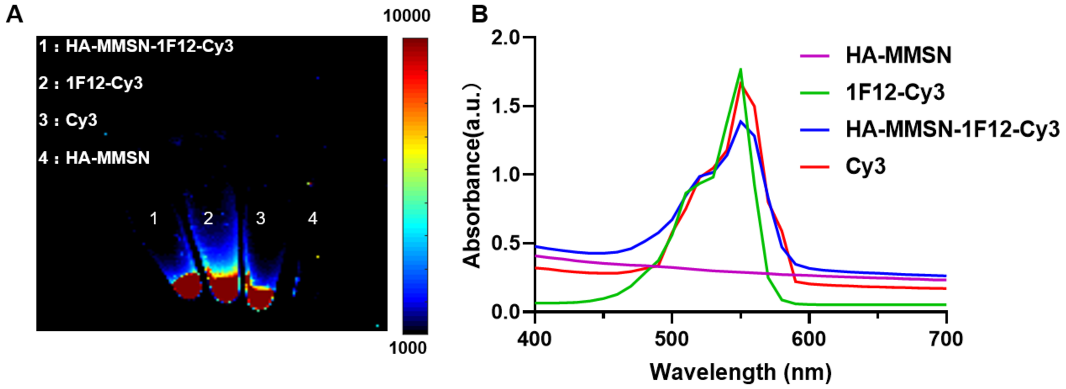

Figure S7. Characterization of Cy3-labeled HA-MMSN-1F12. Fluorescence imaging **(A)** and absorption spectroscopy **(B)** of Cy3, 1F12-Cy3, HA-MMSN-1F12-Cy3, and HA-MMSN.

Supplementary Figure 8

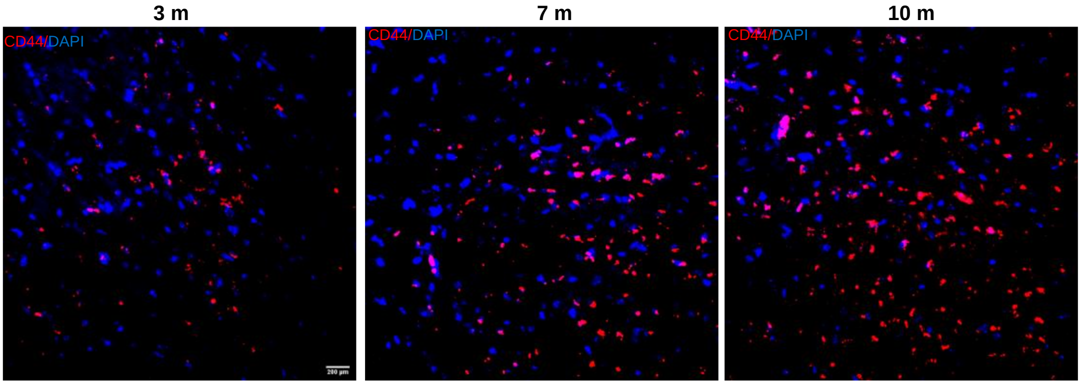

Figure S8. Representative immunofluorescence images of CD44 expression in brain tissue of APP/PS1 mice aged 3, 7, and 10 months, scale bar, 200 μm.

Supplementary Figure 9

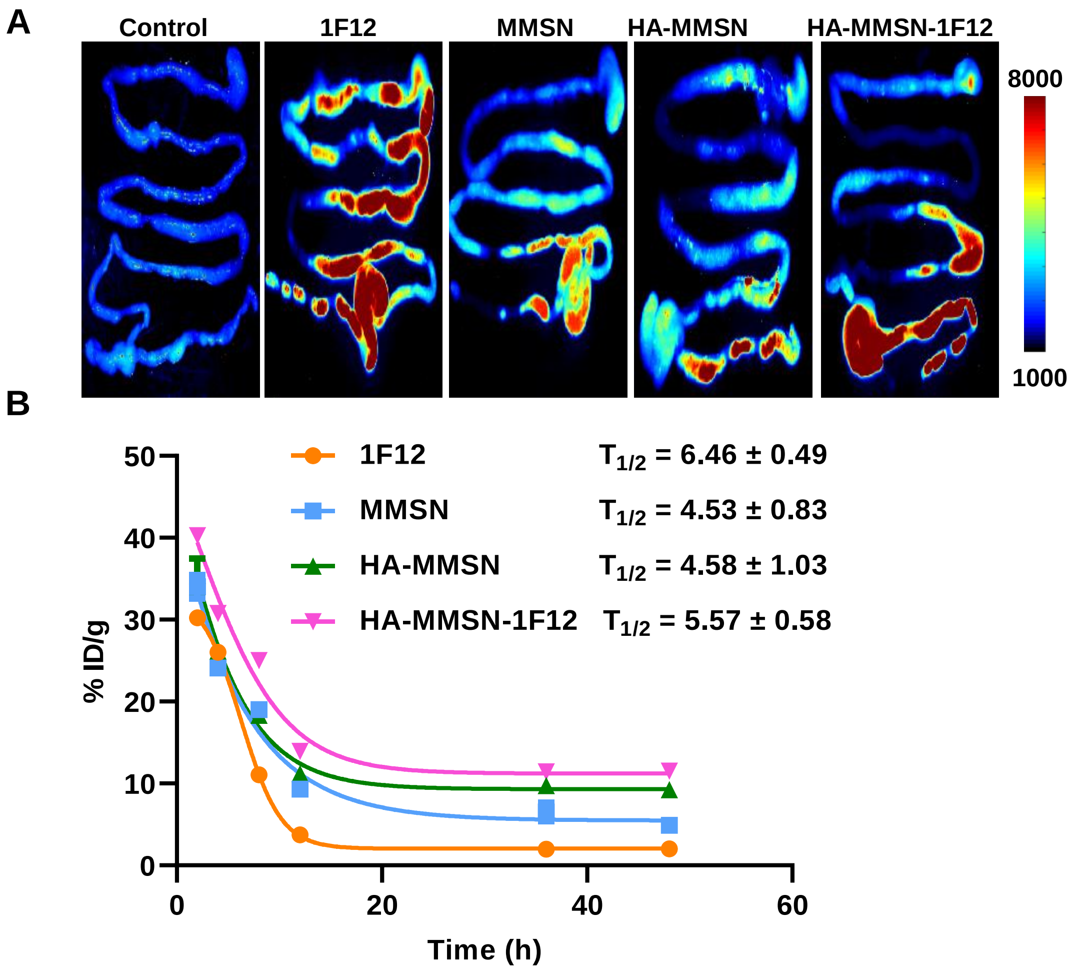

Figure S9. Biodistribution and pharmacokinetics of HA-MMSN-1F12 *in vivo*. (A) Fluorescence imaging of organs from ten-month-old APP/PS1 mice at 6 h postinjection of Cy5-labeled 1F12, MMSN, HA-MMSN-1F12, and HA-MMSN. n = 3 per group. **(B)** Pharmacokinetics of 1F12, MMSN, HA-MMSN, and HA-MMSN-1F12.

Supplementary Figure 10

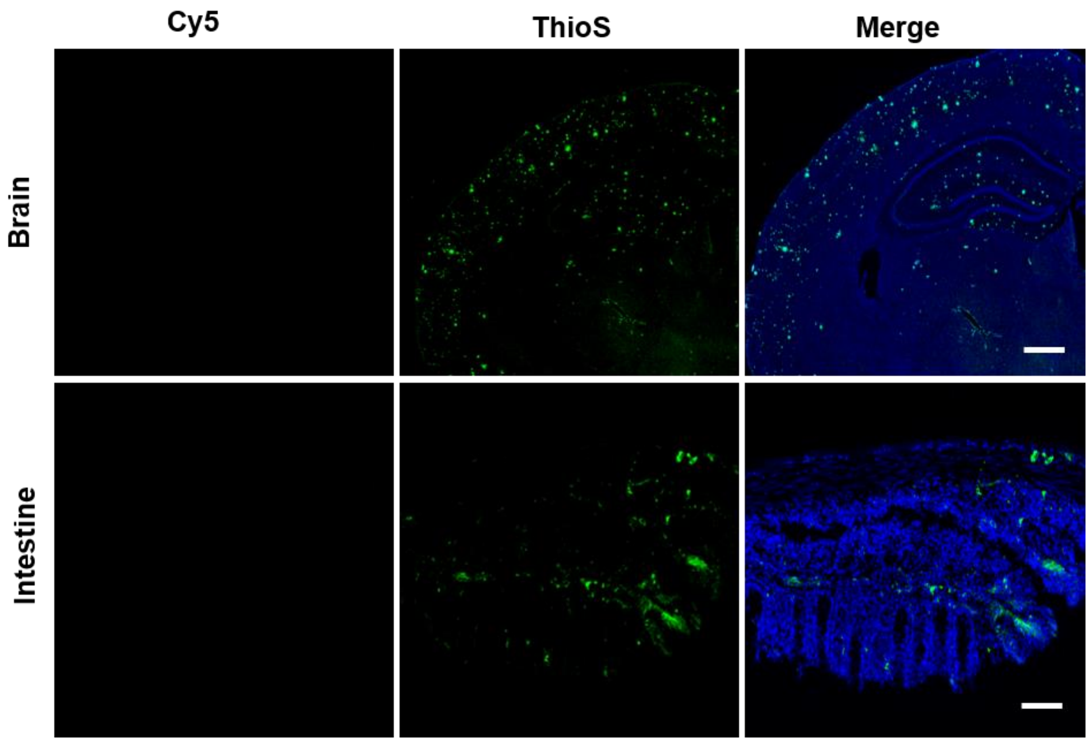

Figure S10. Confocal imaging of tissue sections of the brain and intestine from ten-month-old APP/PS1 mice at 6 h postinjection of HA-MMSN. DAPI (blue), ThioS (Green), and scale bar, 200 µm. n = 3 per group.

Supplementary Figure 11

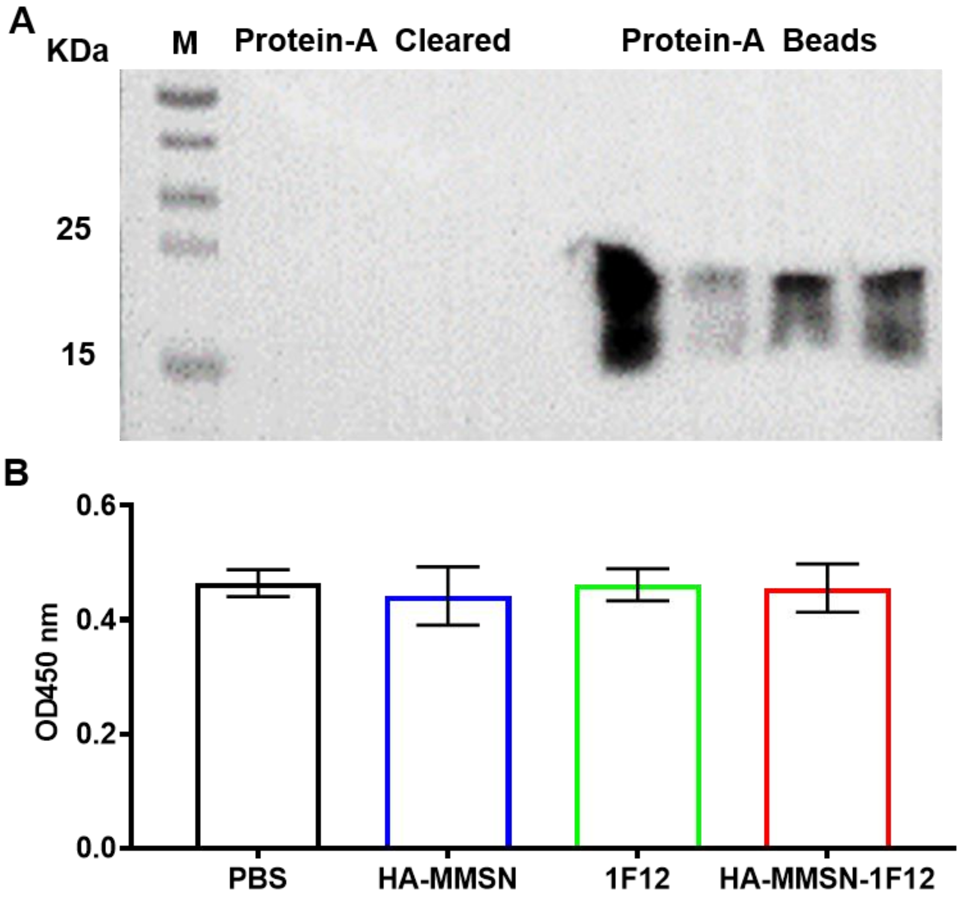

Figure S11. Intravenous administration of HA-MMSN-1F12 clears Aβ from the blood. (A) Evaluation of the ability of intravenously administered HA-MMSN-1F12 to capture all circulating Aβ in peripheral blood. Peripheral blood of APP/PS1 mice after 12 h postinjection of 100 μg HA-MMSN-1F12 was gone through the protein A column. Lanes 1 and 4 were eluates of peripheral blood after passing through the protein-A column; Lanes 5 and 8 were the beads of the protein-A column after the peripheral blood of APP/PS1 mice passed through the protein-A column. **(B)** Ten-month-old male APP/PS1 mice with the closest serum OD450 positive values were selected to reduce errors caused by differences between mice.

Supplementary Figure 12

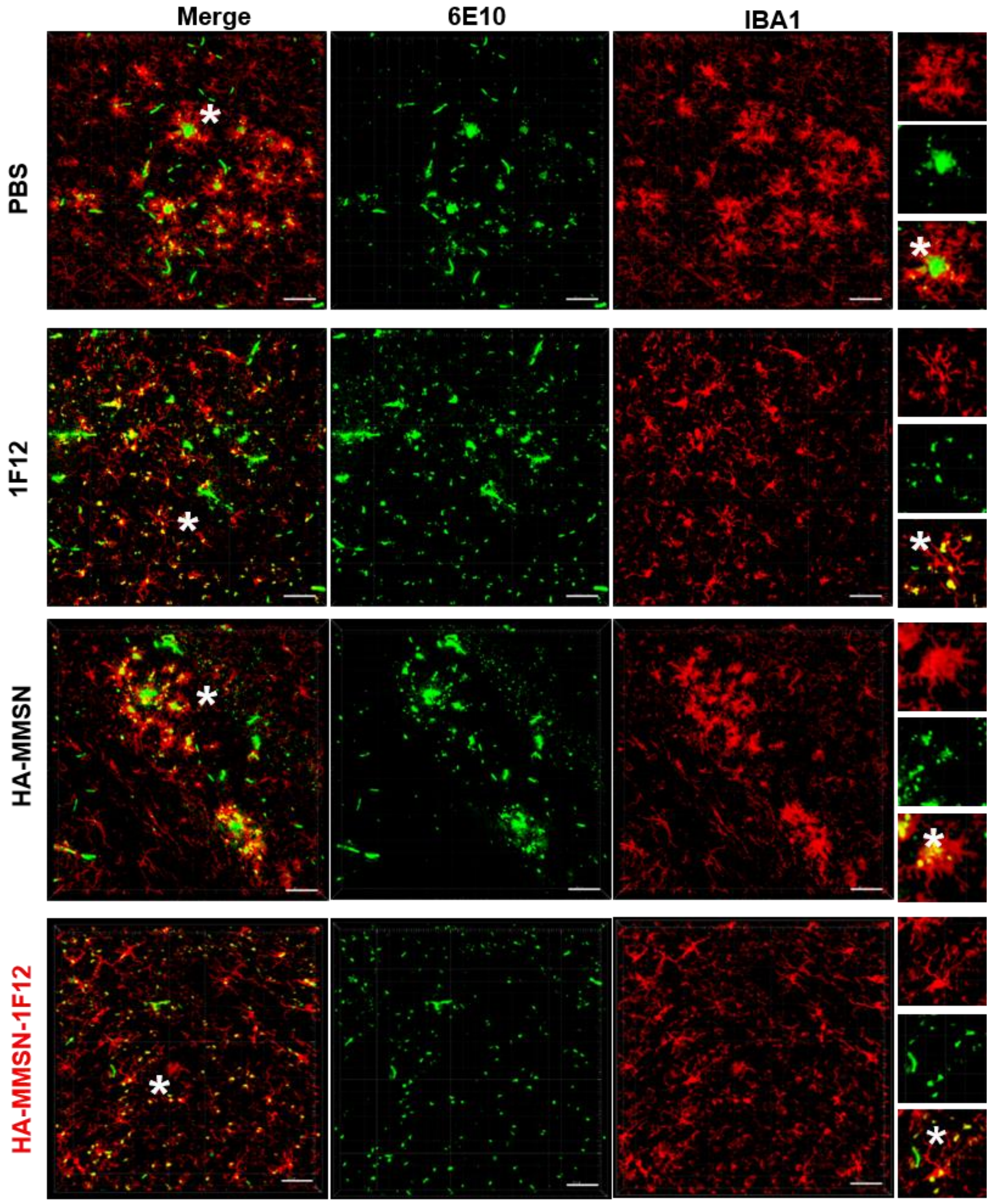

Figure S12. Representative images of microglia and Aβ plaques after immunostaining by anti-IBA1 and anti-Aβ (6E10) in the hippocampal CA1 region of ten-month-old APP/PS1 mice treated with PBS, HA-MMSN, 1F12, or HA-MMSN-1F12. Insets show the morphology of microglia marked with an asterisk (*). IBA1 (red), 6E10 (green), and scale bar, 500 µm.

Supplementary Figure 13

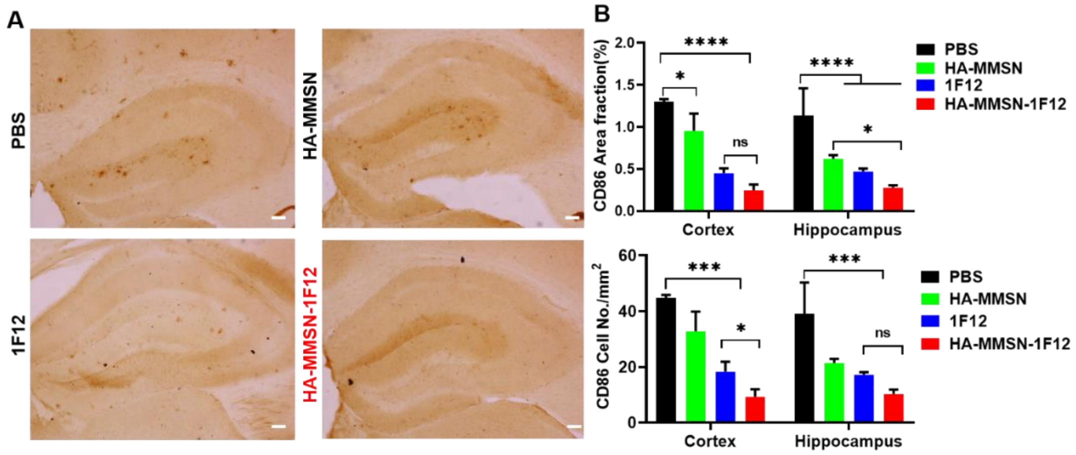

Figure S13. Effects of intravenous injection of HA-MMSN-1F12 on microglia. (A) Immunohistochemical and **(B)** quantitative analysis of activated microglia, scale bar, 200 µm.

Supplementary Figure 14

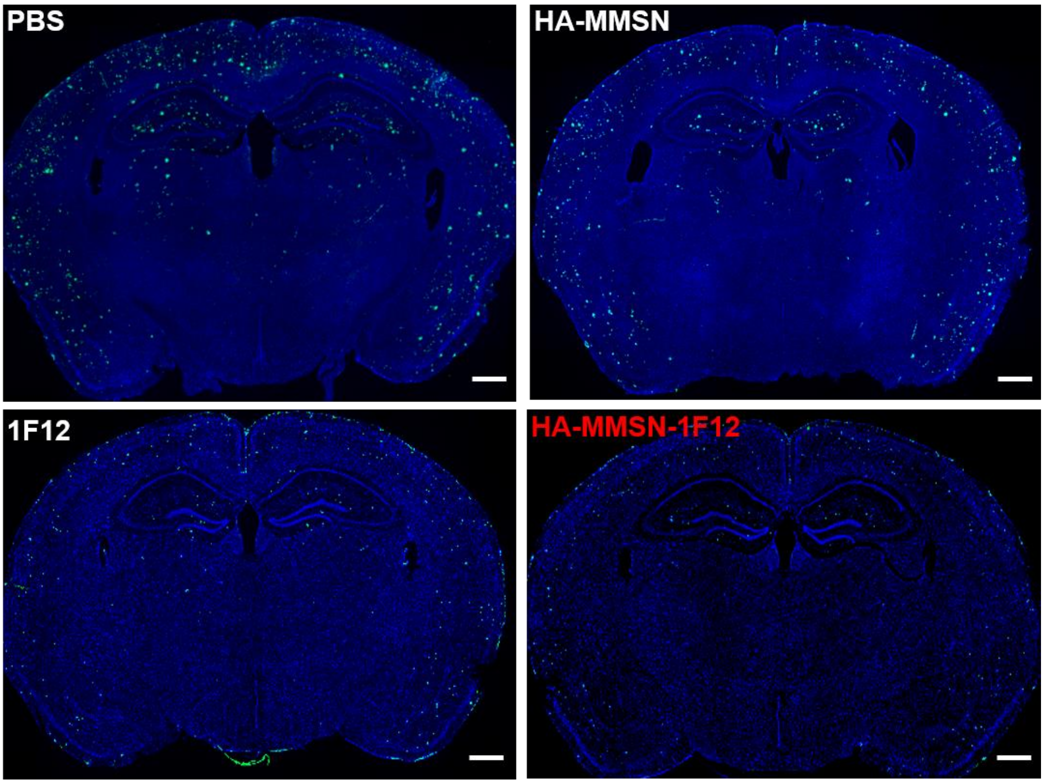

Figure S14. Representative images of ThioS staining in the brain sections of ten-month-old APP/PS1 mice after treatment with PBS, HA-MMSN, 1F12, and HA-MMSN-1F12 (n = 3 per group). ThioS (green) was used to assess the number of Aβ plaques after treatment, scale bar, 200 µm.

Supplementary Figure 15

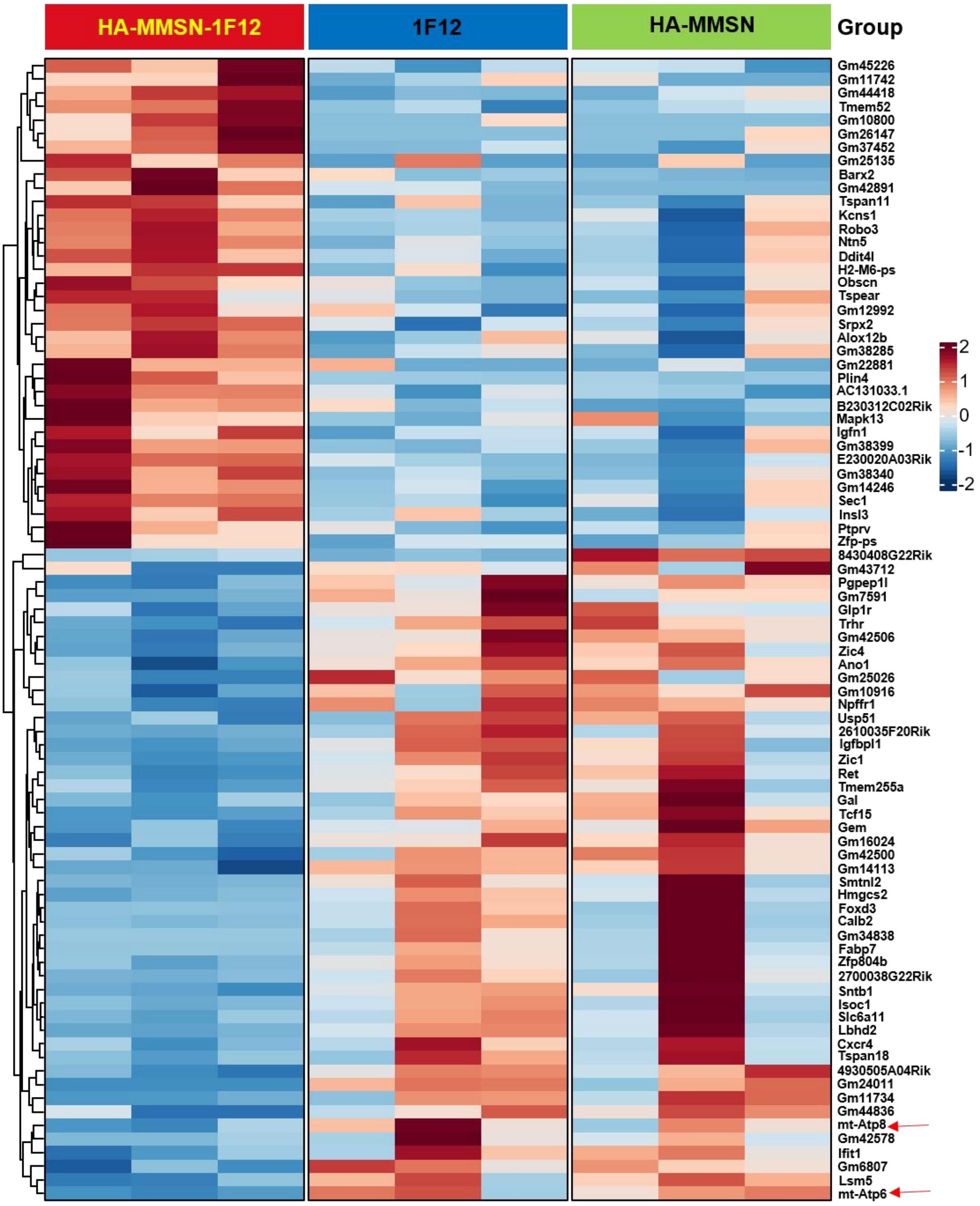

Figure S15. Transcriptome analysis of brain tissues from ten-month-old APP/PS1 mice treated with PBS, HA-MMSN, 1F12, and HA-MMSN-1F12, n = 3.

Supplementary Figure 16

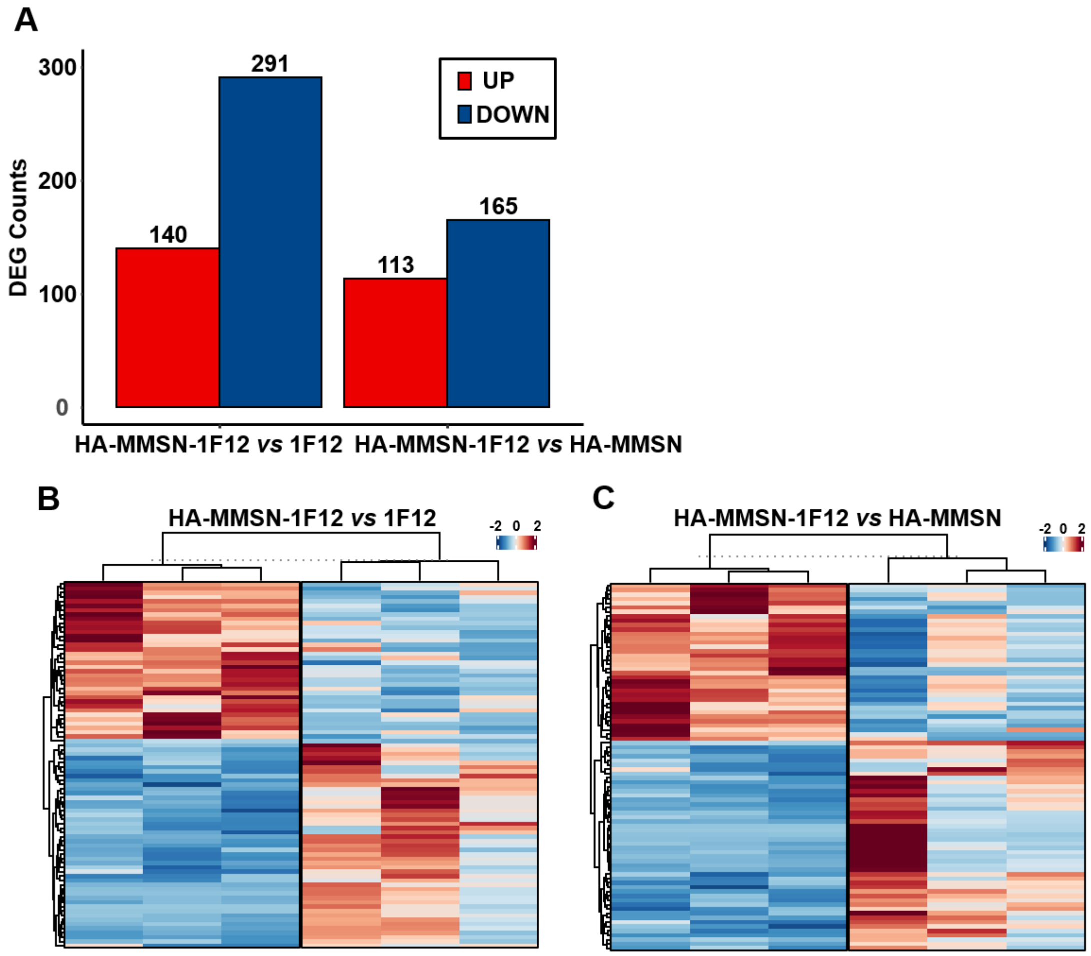

Figure S16. Analysis of DEGs in brain tissues from ten-month-old APP/PS1 mice treated with 1F12, HA-MMSN, and HA-MMSN-1F12. (A) Statistical analysis of DEGs in brain tissues from ten-month-old APP/PS1 mice treated with 1F12, HA-MMSN, and HA-MMSN-1F12. Differential gene expression heatmap between the HA-MMSN-1F12 and 1F12 **(B)** or HA-MMSN **(C)** treatment groups. The sample order revealed hierarchical clustering results, suggesting that the same set of samples had similar gene expression patterns and participated in similar biological processes.

Supplementary Figure 17

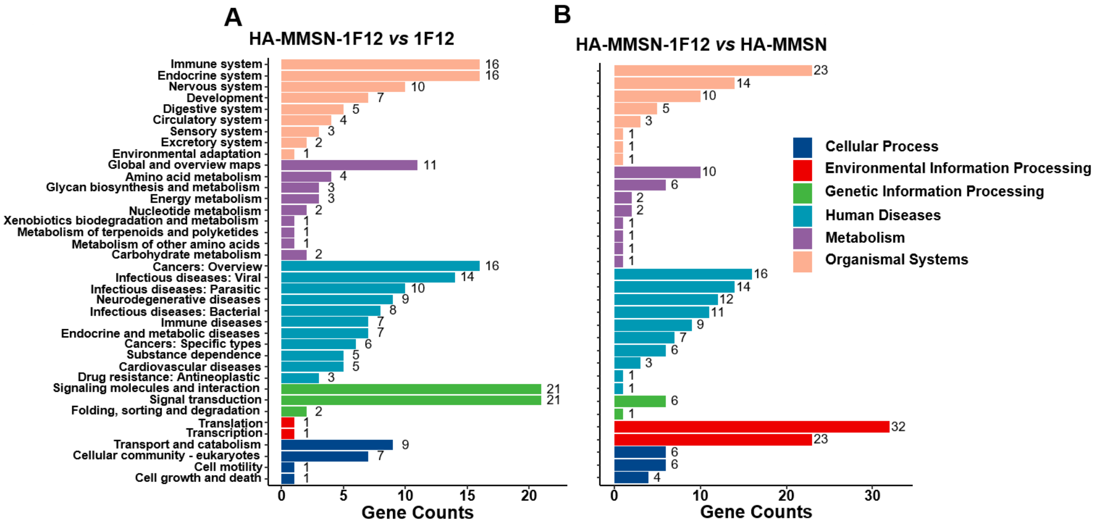

Figure S17. KEGG enrichment bar plot. KEGG enrichment of differential genes between the HA-MMSN-1F12 and 1F12 **(A)** or HA-MMSN **(B)** treatment groups. The ordinate is the name of the KEGG term, and the annotated number above the bar refers to the number of genes enriched by the term.

Supplementary Figure 18

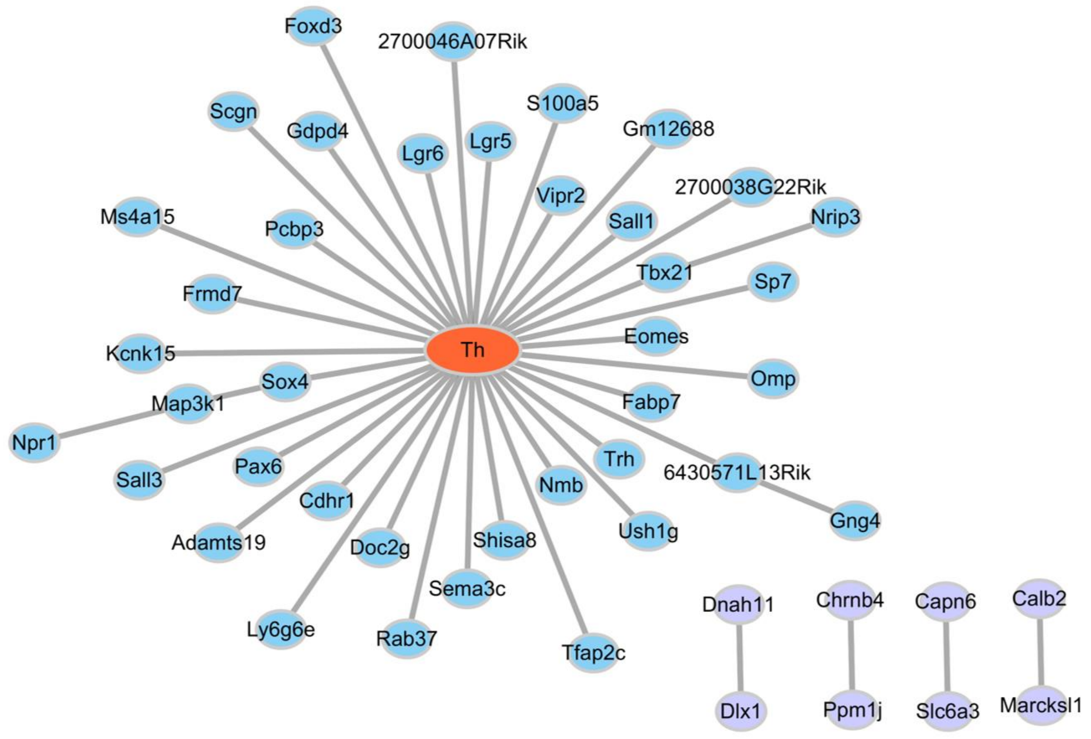

Figure S18. Co-expression network of differential genes related to the immune system, immune diseases, neurological diseases, and neurodegenerative diseases (HA-MMSN-1F12 *VS* 1F12).

Supplementary Figure 19

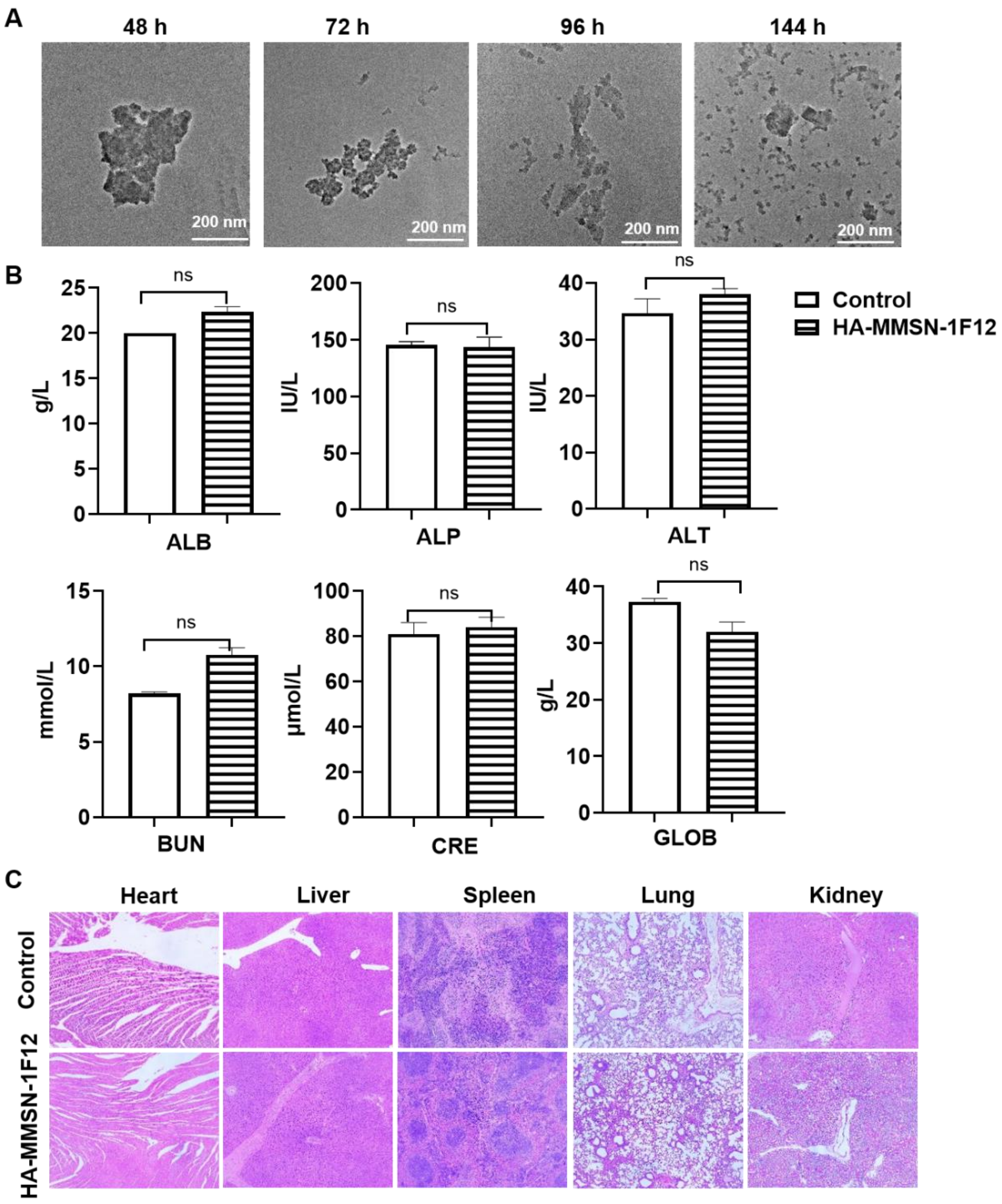

Figure S19. HA-MMSN-1F12 has no detectable side effects in healthy C57BL/6 mice. (A) TEM images of HA-MMSN-1F12 in simulated body fluid (Krebs–Henseleit solution) at 37 °C for 48, 72, 96, and 144 h. **(B)** Blood biochemical analysis of albumin (ALB), alkaline phosphatase (ALP), alanine aminotransferase (ALT), blood urea nitrogen (BUN), creatinine (CRE), and globulin (GLOB) among eight-month-old C57BL/6 mice with or without injection of HA-MMSN-1F12. **(C)** H&E staining of tissue sections of heart, liver, spleen, lung, and kidney from eight-month-old C57BL/6 mice with or without postinjection of 4 mg/kg of HA-MMSN-1F12 per mouse (n = 3

per group), scale bar, 100 μm. The student's t-test (two-tailed) was performed to determine significance. Statistical significance is indicated in the figures by n.s. (no significance).
